# Environmental Impact of Fused Filament Fabrication: What Is Known from Life Cycle Assessment?

**DOI:** 10.3390/polym16141986

**Published:** 2024-07-11

**Authors:** Antonella Sola, Roberto Rosa, Anna Maria Ferrari

**Affiliations:** Department of Sciences and Methods for Engineering (DISMI), University of Modena and Reggio Emilia, Via G. Amendola 2, 42122 Reggio Emilia, Italy; roberto.rosa@unimore.it (R.R.); annamaria.ferrari@unimore.it (A.M.F.)

**Keywords:** fused filament fabrication, FFF, fused deposition modeling, FDM, material extrusion, MEX, additive manufacturing, sustainability, life cycle assessment, environmental impact

## Abstract

This systematic review interrogates the literature to understand what is known about the environmental sustainability of fused filament fabrication, FFF (also known as fused deposition modeling, FDM), based on life cycle assessment (LCA) results. Since substantial energy demand is systematically addressed as one of the main reasons for ecological damage in FFF, mitigation strategies are often based on reducing the printing time (for example, adopting thicker layers) or the embodied energy per part (e.g., by nesting, which means by printing multiple parts in the same job). A key parameter is the infill degree, which can be adjusted to the application requirements while saving printing time/energy and feedstock material. The adoption of electricity from renewable resources is also expected to boost the sustainability of distributed manufacturing through FFF. Meanwhile, bio-based and recycled materials are being investigated as less impactful alternatives to conventional fossil fuel-based thermoplastic filaments.

## 1. Introduction

Additive manufacturing (AM), or 3-dimensional (3D) printing, has progressed from being a rapid prototyping tool to being an industrial mainstream for the production of functional parts. Since objects are built up through the selective addition of material in a layer-wise manner, bespoke or highly complicated geometries can be obtained affordably and effortlessly [1].

It is common sense that AM is “inherently” more environmentally sustainable than conventional fabrication methods because it enables very efficient use of feedstock materials [2]. This is especially true when AM is compared to subtractive technologies like milling and turning, in which the desired geometry is achieved by removing unnecessary material that has to be disposed of [3].

Besides more productive management of materials, AM may afford other environmental benefits over traditional manufacturing. For example, topology optimization may result in lightweight AM parts delivering the same structural performance as conventional components while simultaneously featuring a reduced weight [4,5]. This is expected to cut down on fuel consumption in transportation and aerospace applications [6,7]. Due to the capillary distribution of 3D printers, distributed manufacturing is gradually changing the supply chain, as objects can be produced on demand where they are needed. This reduces the need for physically moving goods while also reducing the space needed for warehouses and deposits. Spare parts can be made available on-the-spot, thus limiting the time required for maintenance of failed or underperforming industrial plants [8]. Finally, assuming that intellectual properties are protected, broken equipment and commodities can be repaired even if legacy parts are not commercially available anymore because the missing components can be reverse-engineered and 3D printed [9].

**Figure 1:**
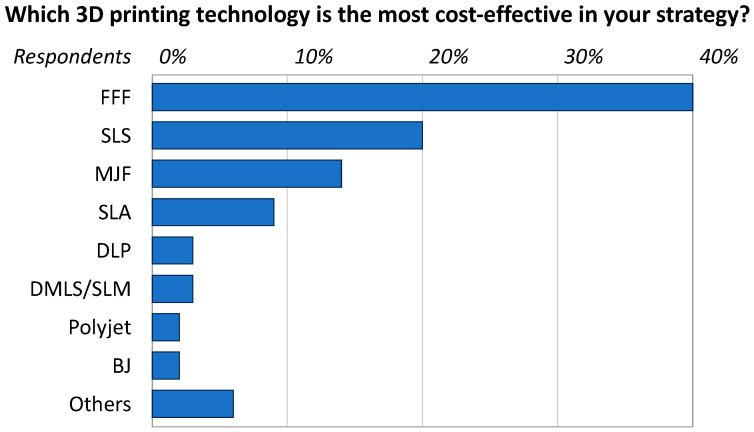
Popularity of AM technologies according to recent statistics (2022). FFF: fused filament fabrication; SLS: selective laser sintering; MJF: multi-jet fusion; SLA: stereolithography; DLP: digital light processing; DMSL: direct metal laser sintering; SLM: selective laser melting; BJ: binder jetting. Adapted from Sculpteo [11].

However, this general understanding is often based on intuitive preconceptions, whereas quantitative evidence would be required to demonstrate the environmental advantages of AM [10]. This is especially true for fused filament fabrication (FFF), which is currently the most widespread plastic-based AM method. As a matter of fact, according to recent statistics summarized in Figure 1, FFF has been flagged as the most cost-effective AM technology for business strategy by 40% of the companies in the field [11]. Thermoplastic filaments are often praised for their recyclability [12], and numerous initiatives are leveraging this advantage, even on an industrial scale. For example, ÉireComposites Teo in Ireland, with the assistance of researchers from the University of Galway, I-Form, and MaREI, have demonstrated that industrial thermoplastic-based composite waste can be recycled into printable filaments [13,14]. Despite this potential material-related benefit, FFF is actually known to be an energy-intensive process, which needs (primary) energy to heat the plastic feedstock above its glass transition temperature or melting point, plus additional (secondary) energy to complete ancillary operations, such as powering up the drive motors moving the printhead around, the ventilation fan at the nozzle exit, and the warm-up system connected to the build platform [15,16,17,18]. FFF is also likely to pose health hazards, such as the release of fine particulates and volatile organic compounds [19,20,21,22].

Life cycle assessment (LCA, sometimes also known as “life cycle analysis”), which is a standardized method for quantifying the potential environmental impacts (EIs) associated with a given product or process, is gaining attention as a reliable tool to fact-check the environmental sustainability of FFF [23,24], also taking into account new bio-based feedstock materials [25] and advanced recycling procedures [26].

The goal of the present contribution is, therefore, to critically discuss the EI of FFF as objectively determined through LCA. To this end, a systematic review was conducted to answer the research question: What is known about the “environmental sustainability” of FFF based on LCA results?

After introducing the functioning mechanisms of FFF in Section 2 and the basic principles of LCA in Section 3, the paper outlines in Section 4 the systematic procedure applied here to interrogate the body of literature. The results of this search are gathered in Section 5, which consists of two main parts. The first part (Section 5.1) provides a survey of the chronology and geography of the available archival papers, while the second part (Section 5.2) delves into the state of the art, captures the environmental advantages and disadvantages of FFF with respect to conventional fabrication technologies and other AM methods, and discusses the importance of adopting new sustainable materials and recycling procedures to limit the EI of FFF. Finally, while keeping in mind that the environmental indicators quantified through an LCA are predictions of impact potentials and not yet facts [27], the conclusions in Section 6 suggest areas of interest for future improvement.

## 2. Functioning Mechanism of FFF

**Figure 2:**
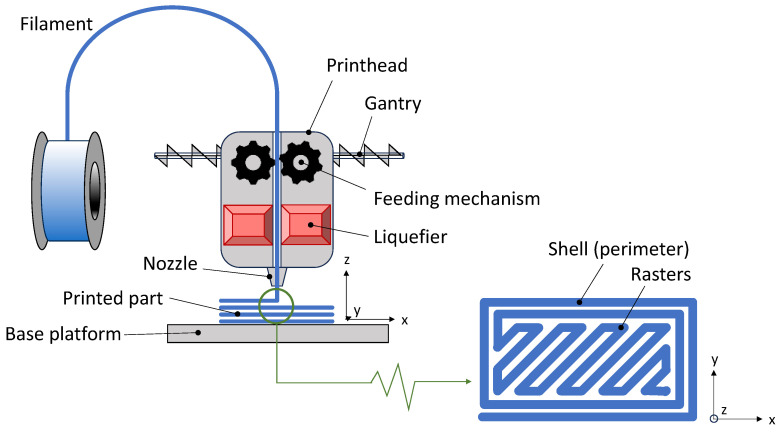
Functioning principle of FFF.

Formally, fused filament fabrication (FFF), also known as fused deposition modeling (FDM), belongs to the material extrusion (MEX) family as per ISO/ASTM 52900:2021 [28]. As the name itself suggests, the peculiarity of FFF is its feedstock, which is a thermoplastic-based filament typically produced by melt extrusion [29]. A schematic representation of the functioning principles of FFF is presented in Figure 2.

Driven by the gears of the feeding mechanism, the filament is pushed into the liquefier, which is the core of the printhead. Here, the feedstock material is heated and melted (or softened for amorphous thermoplastics) and then pushed out of the nozzle. As the molten feedstock is being deposited on the base platform, the printhead, which sits on an “x”-“y” gantry, moves across the plane according to a computer-controlled toolpath. Once the first layer has been completed, the base platform moves downwards (or the printhead moves upwards) along the growth direction, “z”, and then a second layer is added on top of the first one. The process is repeated until the desired geometry is achieved [30]. Three-dimensional objects are thus fabricated layer-upon-layer, where each layer, in its turn, is built up by “rasters” or “strands” of material that fuse together through a sequence of necking, sintering, and interface healing processes [29].

**Figure 3:**
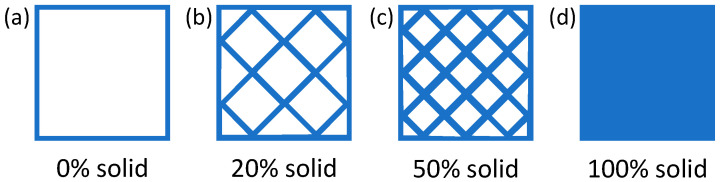
Same part is printed with different infill degrees: (**a**) 0% solid, corresponding to a completely hollow part; (**b**) 20% solid; (**c**) 50% solid; (**d**) 100% solid.

A special trait of FFF is that objects are rarely 100% solid. Conversely, oftentimes, they are largely hollow. While the perimeter of each layer is deposited as a continuous raster in order to provide structural strength and create the visual appearance of a fully solid object, the interior is actually printed with a sparse infill, which may range between 0% (completely hollow part) and 100% (completely solid part), with values around 18–20% being the commonest ones for rapid prototyping [31,32]. The concept of infill degree is illustrated in Figure 3.

**Figure 4:**
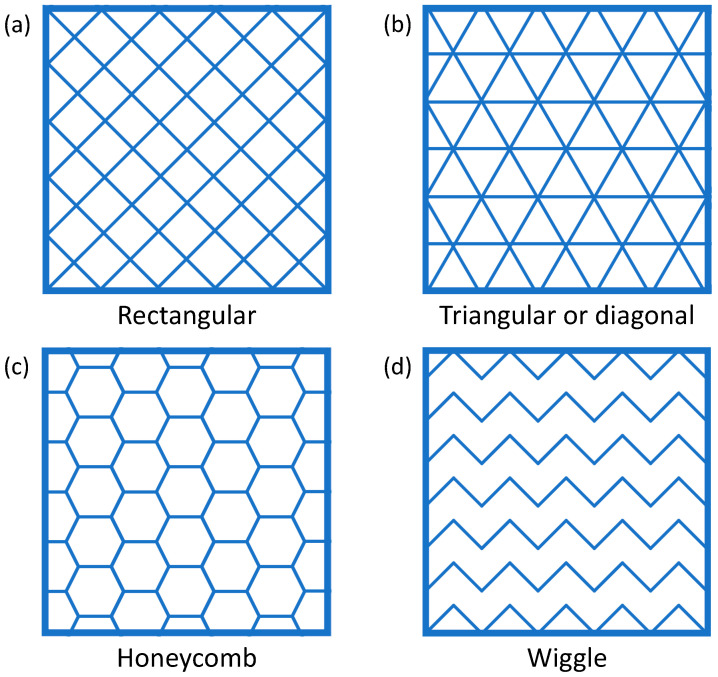
Examples of different infill patterns are (**a**) rectangular; (**b**) triangular or diagonal; (**c**) honeycomb; and (**d**) wiggle.

For the same value of the infill degree, different infill patterns are also possible, and some examples are shown in Figure 4.

Choosing a reduced infill degree must be an informed decision, as this will lower the strength of the printed part. However, it is worth noting that the structural strength (especially under flexural load) does not scale linearly with the infill degree, such that the strength of a part having, for instance, a 20% infill degree is higher than 20% of the strength of the same part having a 100% infill degree [33]. Meanwhile, adjusting the infill degree to the functional requirements makes it possible to print lightweight objects in a shorter time than fully solid parts. 

## 3. Basic Principles of LCA

Life cycle assessment (LCA) is a methodology that quantifies the potential EI of a given product throughout its lifespan, where “product” may refer to goods, processes, and services [34]. Nowadays, LCA is rigorously conducted in compliance with international standards, mainly ISO 14040:2006 [35] and ISO 14044:2006 [36]. The European Commission is also developing the Environmental Footprint initiative, which outlines an up-to-date LCA methodology to assist companies wishing to market their products as environmentally friendly in the European Union [37].

**Figure 5:**
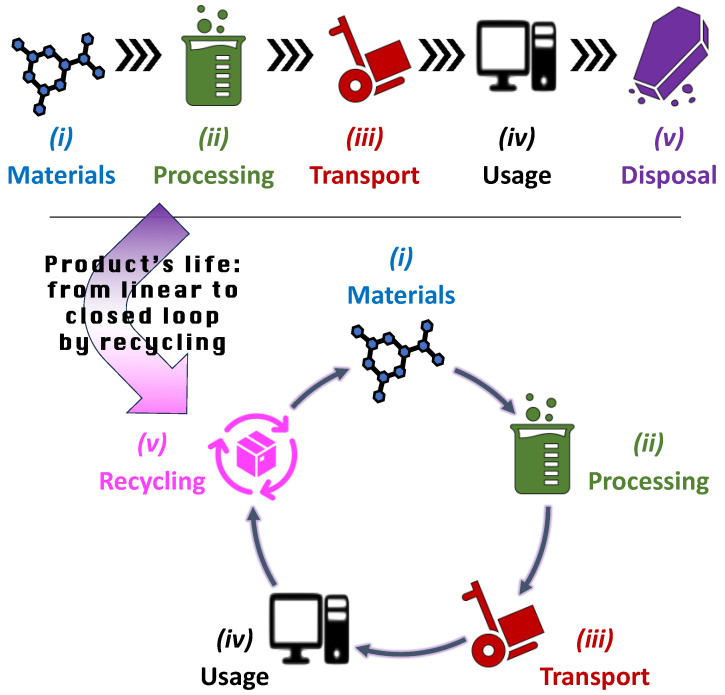
Five stages of a product’s life according to the LCA methodology. If “disposal” is replaced by “recycling”, the product’s life trajectory changes from linear to circular.

As illustrated in Figure 5, in order to complete an LCA, a product’s life is schematically described through five stages: (i) raw material extraction, which is the so-called “cradle” of the product; (ii) manufacturing and processing; (iii) transportation; (iv) usage and retail; and (v) disposal, which is the so-called “grave” [38]. Although goods are likely to be transported several times along the supply chain, “transportation” is conventionally placed between “processing” and “usage” to highlight the shift from factory to customer.

This representation of a product’s life is linear, as it goes from input materials as the start point to disposal of the used product as the end point. For this reason, it is generally defined as “cradle to grave”. However, more and more often, the disposal stage is being replaced by recycling, which converts the waste product (or its components) into new input materials, thus changing the product’s life from a linear segment to a circular trajectory along a closed loop, sometimes referred to as “cradle to cradle” [39]. Driven by the “circular economy” principles, the number of case studies that make use of LCA in order to verify the advantages of recycling is increasing [40], with examples that come from a variety of industrial sectors, from plastic packaging [41] to photovoltaic cells [42], from automotive lithium-ion batteries [43] to aggregate concrete [44,45], from electronics [46] to nutrients embedded in wastewater [47].

Besides the “cradle to grave” and the “cradle to cradle” models, other LCA approaches are also feasible. For example, “cradle to gate” assessments are focused on the first two stages of the product’s life, namely materials and processing, which occur prior to transportation to the consumer (this model still includes the transportation of raw materials from the extraction site to the processing plant). “Cradle-to-gate” analyses are especially useful for the quick assessment of internal processes [48].

**Figure 6:**
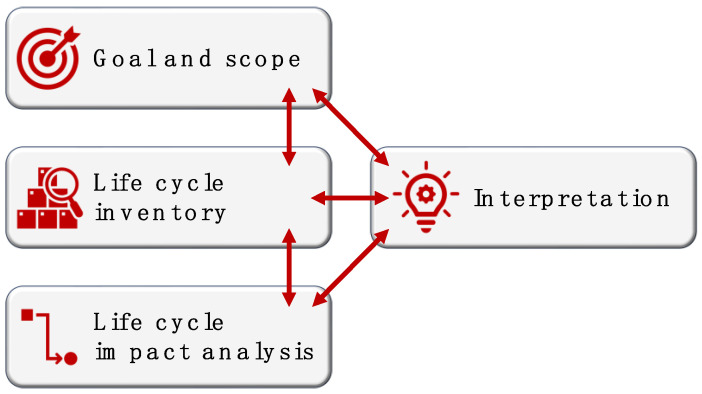
Four stages are required to complete an LCA.

According to Figure 6, the LCA of a product or system is typically completed through four mutually related stages, namely: (i) goal and scope definition; (ii) life cycle inventory (LCI); (iii) life cycle impact assessment (LCIA); and (iv) interpretation of the results [34,49,50,51].

The starting point of any LCA study consists of defining the goal and scope, which means discriminating between what will be analyzed and what will not. Firstly, it is necessary to identify the “functional unit” of the LCA, which is the reference unit to which all the obtained EIs will be referred [27]. Setting the analysis boundaries implies choosing the appropriate LCA model, be it “cradle-to-gate”, “cradle-to-grave”, “cradle-to-cradle,” or any other model that is relevant to what the company (or other relevant stakeholders) wants to ascertain. In this initial step, the analyst should also plan what kind of “environmental impact(s)” the product will be assessed against during the LCIA step, thus defining the so-called “impact categories.”

The LCI step is necessary to gather all the required information to be used for the impact assessment. Working within the boundaries outlined in the “goal and scope” definition stage, the LCI looks at all the inputs and outputs associated with the product’s (or service’s) life cycle. Wherever possible, data should be obtained from the corresponding departments within the company or directly from the suppliers and distributors of the value chain. This is defined as “primary data” (or “foreground information”). Oftentimes, real figures may be unavailable. If this is the case, average values pertaining to the same industry sector can be used instead. This is defined as “secondary data” (or “background systems”).

The LCIA is the elaboration stage, wherein the data acquired through the LCI is linked to different impacts on the environment, conveniently converted to equivalent units for calculation and comparison purposes, and finally translated to output parameters that can also be normalized or weighted (“valuation”) as needed.

“Interpretation” is conventionally positioned last because it becomes particularly meaningful once the results of the LCIA become available. However, understanding, analyzing, and questioning are necessary through each stage of the assessment.

## 4. Systematic Review: Protocol

As mentioned above, this section is primarily methodological, as it provides a detailed account of the procedures applied in the present systematic review. The results are reported and discussed in Section 5.

**Figure 7:**
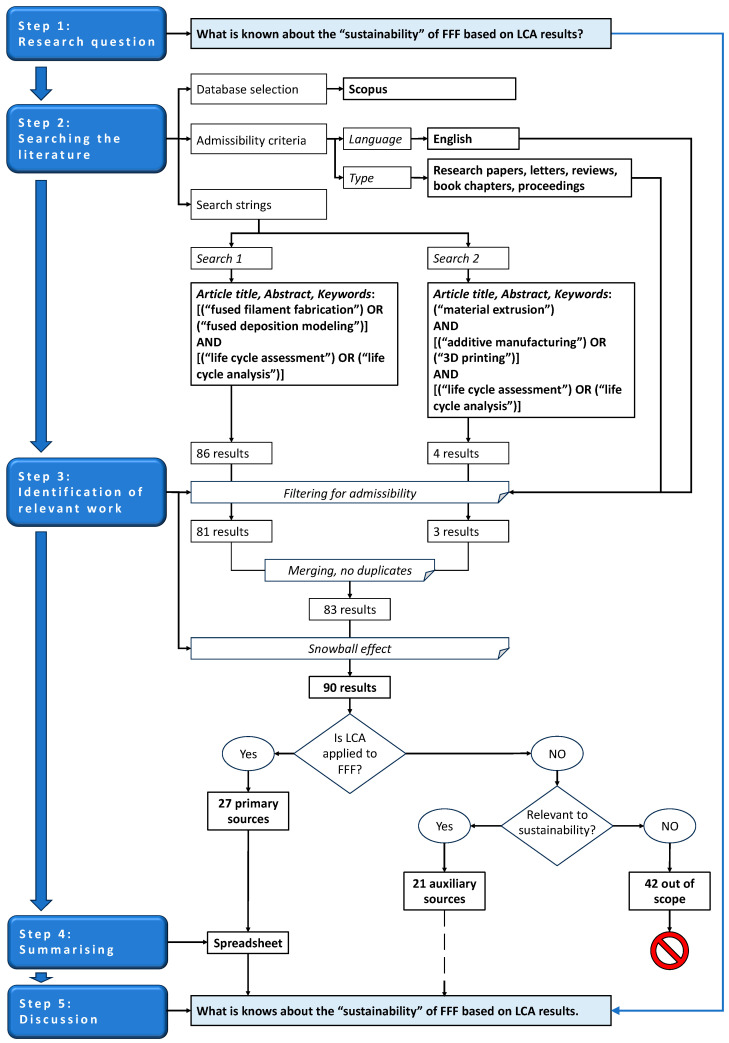
Workflow followed in this systematic review.

A systematic review is an analysis of the literature conducted according to pre-defined eligibility criteria in order to answer a given research question in an unbiased way [52,53]. The five steps that guided the systematic review in this contribution are detailed in the following paragraphs, and the complete workflow is schematized in Figure 7.

The protocol followed in this literature review adhered to the transparency and repeatability criteria underpinning the PRISMA statement [54,55]. However, with the PRISMA approach being primarily intended for clinical studies, some methodological changes were deemed necessary to accommodate the different topics being investigated here. Moreover, as further explained in the following paragraphs, two separate searches had to be conducted in the same database in order to account for the different names routinely applied to FFF.

### 4.1. Framing the Question (Step 1)

Our intention with this systematic review was to reply to the question: What is known about the “sustainability” of FFF based on LCA results?

### 4.2. Searching the Literature: Database, Admissibility Criteria, and Search Strings (Step 2)

In order to answer the abovementioned research question, a literature survey was conducted in Scopus. This archive was chosen among others because it only indexes content that has been assessed by an independent review panel of experts in their respective fields. Meanwhile, it offers wide coverage, including (at the time of conducting this research) more than 26,000 serial titles and 243,000 books [56].

While searching the literature, two admissibility criteria were defined based on (i) the language and (ii) the publication type. Firstly, it was decided to only consider contributions written in English as the standard language of scientific literature. Secondly, experimental papers (including both short communications and full-length articles), letters, reviews, book chapters, and proceedings were the only accepted document types. Letters and review papers were deemed relevant as they may present case studies and redirect to additional literature through cross-referencing. Book chapters are sometimes regarded as “gray literature” because they are not peer-reviewed. Nonetheless, they were not excluded because they must meet numerous requirements to be indexed in Scopus [57]. Likewise, proceedings must also satisfy numerous parameters to be indexed in Scopus. Sometimes, proceedings are additionally peer-reviewed prior to publication under the responsibility of the organizing committee of the conference they have been presented at.

Scopus was interrogated on 3 October 2023 in two stages. A first search was run using the specific name of the technology, either “fused filament fabrication” or “fused deposition modeling.” The search string was therefore:

*Article title, Abstract, Keywords*:

(“fused filament fabrication”) OR (“fused deposition modeling”)

AND

*Article title, Abstract, Keywords*:

(“life cycle assessment”) OR (“life cycle analysis”)

A second search used the standardized name “material extrusion.” However, in order to disambiguate the AM technology from the production of extrudates (films, wires, cables, etc.) by melt processing, the search string was modified to include “additive manufacturing” or “printing,” such that the search string became:

*Article title, Abstract, Keywords:*

(“material extrusion”)

AND

*Article title, Abstract, Keywords:*

(“additive manufacturing” OR “printing”)

AND

*Article title, Abstract, Keywords:*

(“life cycle assessment” OR “life cycle analysis”)

### 4.3. Identification of Relevant Work (Step 3)

The first search returned 86 items. Within this preliminary pool, one contribution [58] was written in Korean, and since no official translation in English could be found for it, it was left out. Four more results were discarded because they were mere lists of proceedings’ titles.

The second search returned four items. One contribution (the same one already identified in the first search) was written in Korean, and hence it was disregarded. The remaining three items were admissible.

When the results of the two searches were merged, one duplicated item was discarded, thus leading to a list of 83 items. Meanwhile, seven additional works of potential interest were retrieved through the snowball effect (cross-referencing).

Ultimately, 90 items were moved over to a more refined assessment that evaluated the relevance of their content by reading the title, abstract, and keywords. In doing this, for a matter of practicality, the overarching problem of this systematic review, as expressed in Step 1, was broken down into sub-questions starting from the fundamental point: did the authors analyze the EIs of FFF via LCA? If yes, the contribution was classified as a “primary source” and hence further investigated by reading the full text in order to respond to the following inquiries: Why did the authors perform this LCA? What did they analyze? How did they conduct their LCA? And what did they find? If not, the subsequent question was: Is the contribution still useful to gain a deeper understanding of the EIs of FFF? If yes, the contribution was read and then used as an “auxiliary source” for discussing the findings of the primary sources (i.e., the papers presenting an LCA of FFF, as explained above). If not, the contribution was classified as “out of scope” and hence discarded because it was not relevant to the problem addressed by this systematic review.

Owing to the “yes/no” selection criteria applied, the identification of the primary sources was straightforward and hence conducted by one author only (A.S.). The screening for the auxiliary sources was completed by two authors (A.S. and R.R.) separately. The results were then compared, and only those sources that had been classified as not relevant to environmental sustainability by both assessors were defined as “out of scope”.

### 4.4. Summary of the Relevant Literature (Step 4)

A summary of the primary sources was arranged in a spreadsheet that accounts for both the bibliographic information and the LCA details of each contribution.

In terms of bibliographic information, the spreadsheet lists the authors’ names, the paper’s title, the year of publication, the journal/source, the nationality of the first author, the nationality of the corresponding author, and the total number of countries involved.

As regards the LCA, the spreadsheet includes the scope (in response to the sub-question: “Why did the authors perform this LCA?” see Section 4.3); the feedstock material used for printing, the boundaries, and the functional unit (sub-question: “What did the authors analyze?”); the LCA procedure, including, wherever available, the database used for retrieving the background data in the LCI stage and the method applied to determine the EIs in the LCIA stage, together with the LCA software employed (sub-question: “How did the authors conduct their LCA?”); the main outcomes and limitations of the LCA (sub-question: “And what did the authors find?”).

The spreadsheet, which must be considered an integral part of this systematic review, can be found in the Supplementary Information (Table S1).

In this file, all primary sources were categorized into four groups, as discussed below in Section 5.2; the contributions within each group were listed in alphabetical order based on the first author’s family name. It is worth noting that the spreadsheet gathers information regarding the LCA procedure solely for the sake of completeness, meaning that this summary does not imply any judgment about the methodological exactness of the LCA, although there appears to be a noticeable disparity in accuracy across the literature.

### 4.5. Critical Analysis (Step 5)

The primary sources were analyzed in order to figure out the geography, the temporal evolution, and the main stakeholders (journals) involved in the environmental assessment of FFF. The results are presented and discussed in Section 5.1. As the main scope of this systematic review, great attention was then paid to what LCA has revealed so far about the environmental sustainability of FFF (Section 5.2).

Auxiliary sources were leveraged to better comprehend the evidence provided by the primary sources, and where needed, additional literature was also referenced to this end.

As previously mentioned, “out of scope” contributions were not analyzed any further, as they were deemed irrelevant to this systematic review. Nonetheless, it may be useful to point out that most “out-of-scope” contributions dealt with the fatigue behavior of FFF parts, which is a clue of a similarity (and, possibly, an ambiguity) in terminology.

## 5. Results and Discussion

### 5.1. Statistics and Demography

**Figure 8:**
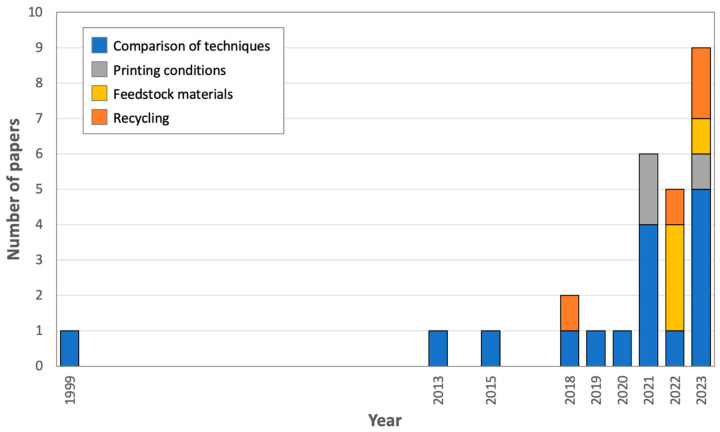
Evolution over time of the literature dedicated to the LCA of FFF.

According to the data available in the Supplementary Information, the identified primary sources summed up to 27 contributions. Notably, as shown in Figure 8 (depicting the evolution over time of the scientific literature devoted to LCA and FFF), one ground-breaking paper was published by Luo et al. [59] in 1999, even before international standards had been issued to regulate LCA. Then, renewed attention has been paid to the environmental assessment of FFF since 2013. However, most of the literature has been published over the last 3 years, with 6 contributions in 2021, 5 in 2022, and 9 in 2023 (until the end of October), thus making 20 contributions out of 27 (74%).

This increasing popularity can be attributed to several factors acting simultaneously. On the one hand, the environmental crisis has been attracting much attention in recent years. The growing awareness that natural resources are limited [60] and that climate change is already impacting our world [61] has called for the identification of mitigation strategies. Prompted by the high levels of public concern, governments are now pushing towards the adoption of greener manufacturing practices. Meanwhile, FFF has gained momentum after Stratasys’ patents expired in 2009. The more flexible conditions on intellectual properties and the diffusion of open-source FFF printers have since sparked research in new materials and systems [62,63], favored the investigation of the processing-structure-properties-performance (PSPP) relationship [64], and ultimately led to an increasing interest in the potential societal challenges that may accompany the wider uptake of this technology, including its environmental footprint [65]. It should also be mentioned that, in November 2017, the Journal of Industrial Ecology published a Special Issue on “Environmental Dimensions of Additive Manufacturing and 3D Printing,” which initiated a critical discussion on the methodology being used and results being obtained for the environmental assessment of AM, including FFF [66].

As further discussed in the following paragraphs, it is possible to identify some thematic categories within the broader framework of the LCA of FFF. Interestingly, while attention to the EI of FFF as compared to other fabrication techniques—either “conventional” or “additive”—has remained constant over time, a growing number of contributions have been dedicated to understanding how the EI of FFF can be reduced through the appropriate tuning of the printing parameters, the choice of greener feedstock materials, or the implementation of recycling procedures. This may be a hint that mitigation strategies are sought after in order to remediate potential criticalities being perceived in the environmental performance of FFF.

Further insight into the body of literature may be gained by considering the demographic data extracted from these primary sources. As shown in the Supplementary Information, the country of origin is nearly always the same for the first author and for the corresponding author. Similarly, just a few contributions required the involvement of scientists from different countries (four contributions from two countries and only one contribution from three countries). This suggests that international collaborations play a minor role in advancing research in the field, likely because the results of LCA (especially through the LCIA and interpretation stages) are deeply influenced by spatial variation and local environmental uniqueness [51]. With a focus on the corresponding author as the representative (contact person) for each contribution, Italy clearly dominates the panorama, with 7 papers out of 27. The European Union (EU) as a whole makes for 10 contributions (including, in alphabetical order: Belgium, 1; Germany, 1; Italy, 7; and Malta, 1), which correspond to more than one-third (37%) of the available literature. This is consistent with the European vision of a circular economy as a pillar that will “modernize the EU industrial base to ensure its global competitive edge and preserve and restore the EU’s natural capital” [67]. Plastic waste management, in particular, was identified as a priority area in the Circular Economy Action Plan released in 2015, which was then followed by the Strategy for Plastics in a Circular Economy issued in 2018. The EU strategy strongly promotes plastic recycling (especially packaging) to reduce the amount of plastic litter [68], while also strengthening the role of science to prevent the environmental and health risks associated with microplastics, for example, through the adoption of biodegradable plastics [69].

Finally, it is worth considering that the LCA of FFF is a cross-disciplinary theme. This is clearly mirrored by the journal titles listed in the Supplementary Information, which pertain to manufacturing, materials, and sustainability.

### 5.2. LCA Outcomes

As previously mentioned, research in the EI of FFF through LCA can be broadly classified into four themes, namely (i) the comparison between FFF and conventional technologies (mainly injection molding) and between FFF and other AM methods (blue highlight in the spreadsheet in the Supplementary Information); (ii) the effect of the printing parameters on the EI of FFF (gray highlight); (iii) the role of different feedstock materials (yellow highlight); and (iv) the potential advantages associated with recycling (orange highlight).

#### 5.2.1. How Does FFF Compare to Other Manufacturing Technologies?

##### Conventional Manufacturing Technologies

Many primary sources in the literature compare the EIs of FFF to those of other manufacturing technologies, either “conventional” or “additive.” However, the outcomes are often mutually contradictory, depending on the adopted functional unit and the selected processing conditions. Moreover, the results of an LCA are only applicable within the geographical and technological context in which they were modeled [27]. Oftentimes, as further discussed in the following paragraphs, the main contribution to the EI of FFF originates from energy consumption. However, energy consumption is both machine-specific [59] and case-specific [70]. This leads to different results when comparing the EI of FFF to conventional technologies.

According to Faludi et al. [71], the sustainability of AM as compared to computer numerical control (CNC) machining largely depends on the percent utilization of each machine. This was demonstrated for both FFF and inkjet printing, which was also included in the LCA. When producing two parts, one presenting a complicated geometry (as often seen in AM) and one featuring simple plane surfaces and straight holes (as often made by CNC machining), the FFF printer scored the lowest EIs per part at both high and low utilization. However, the LCA did not account for post-processing, which might be needed to improve the surface finish of FFF parts up to the quality of inkjet or CNC parts. The inkjet printer sometimes performed better and sometimes worse than the CNC system, depending on the idle time and the applied process parameters. Electricity use was identified as the main reason for the impact of both AM methods. The same happened with CNC machining at low utilization, but material waste became dominant at high utilization, with the contribution of cutting fluid being comparable to electricity use. Notably, under the hypothesis of maximal utilization, fossil fuel depletion and climate change remained the dominant types of ecological damage for all manufacturing methods because the production of plastics (which had the worst impact on CNC machining due to the large volume of plastic waste) and the average production of electricity in the U.S.A. (which had the largest impact for FFF and inkjet printing) are both fossil fuel-based activities.

Reddy et al. [72] explored the sustainability of FFF in the flourishing field of personalized medicine. With respect to conventional tableting procedures, FFF had a minimal adverse impact on the environment. Specifically, CO_2_ emissions (associated with global warming), human toxicity, water consumption, and impact on fossil and mineral resources were lower for FFF because 3D printing required fewer processing steps than conventional tableting. Moreover, FFF drastically limited material waste and mostly used safe chemicals such as polyvinyl alcohol (PVA) impregnated with ascorbic acid (vitamin C). Conversely, high carcinogenic susceptibility was attributed to conventional tableting due to the chemicals routinely used for processing sucrose and dextrose.

Garcia et al. [73] estimated the “Global Warming Potential” (GWP) and the “Cumulative Energy Demand” (CED) associated with FFF and with injection molding when a dumbbell-like specimen complying with ASTM D638 was produced in batches of 7, 14, 50, and 100 units. The LCA demonstrated that both the GWP and the CED of injection molding decreased with increasing batch size due to the reduced amount of energy required for each part until they plateaued out for batches exceeding 50 units. As for FFF, the main contributions to GWP came—in order—from material used, base platform heating, and printing operations, while the main contributions to CED were due to base platform heating and printing operations, with the impact of material use being negligible. However, as acknowledged by Garcia et al. [73], base platform heating in this LCA accounted for the greatest part of the EI associated with FFF due to the fixed heating time of the printer in use, which could not be reduced between subsequent jobs, although the base platform was already hot. Notably, both the GWP and the CED of FFF decreased linearly when the infill degree was reduced because less material and less energy were needed for printing each part. Regardless of the infill degree, the EIs were lower for FFF than for injection molding when small batches of 7 and 14 samples were produced, but injection molding became less impactful for larger batches of 50 and 100 parts. Meanwhile, Garcia et al. [73] also noticed that the ultimate tensile strength (UTS) of the FFF parts decreased with decreasing infill percentage and that, even for 100% infill degree, the tensile strength remained lower than that of the injection-molded parts by nearly 19%. In order to account for the interplay between EI and mechanical strength, the mechanical eco-efficiency was thus defined as the UTS-to-GWP ratio. Notably, this indicator for FFF increased with increasing infill degree, signifying that the increase in mechanical strength associated with a higher infill degree was able to outweigh the increase in GWP.

The role of the production volume is the main research question explored in the contribution by Bezzina and Refalo [70], who compared FFF to injection molding for mass-scale fabrication of 1.5 million cosmetic packaging boxes (“compacts”) yearly for 12 years, where 12 years was the expected lifetime of the injection molding machine system, against 5 years expected for a typical FFF printer. For comparative purposes, the EIs were quantified through the “human health” and the “ecosystem” endpoints. It was found that the potential impact on human health prevailed over the potential impact on the ecosystem for both technologies. However, FFF produced a five-fold greater EI than injection molding on both endpoint categories, with printing energy consumption generating 80% of the impact. This was the result of the extremely long time required for printing each compact since the cycle time for FFF was 5 h, against 7 s required for injection molding. In the case study proposed by Bezzina and Refalo [70], the functional unit was defined according to the real needs of a company whose business is the mass production of plastic packaging. However, it may be arguable that adopting FFF for printing 1.5 million relatively simple, identical parts per year would be a sensible decision. In the contribution by Bezzina and Refalo [70], this led to the (somewhat paradoxical) conclusion that 2170 printers would be needed to meet the yearly production volume. Considering the expected lifetime of 5 years, 6510 FFF printers would thus be required over the next 12 years. Conversely, 4.45 million compacts could be produced annually just using two injection molding machines, one for the lid and one for the base of the compact. Not surprisingly, the total cost for producing each compact was also much higher (17 times) using FFF (EUR 1.58) than using injection molding (EUR 0.09). This difference was largely due to the feedstock material since ABS filaments are more expensive than pellets (indicatively, 30 EUR/kg against less than 2 EUR/kg). Due to the lengthy printing operations, labor, machinery, and energy consumption were also more expensive for FFF. These results seem to contradict the general understanding that FFF can afford substantial cost benefits because parts can be produced without any tooling. Indeed, FFF printers are generally more affordable than injection molding machines, but their productivity is much lower. To clarify, in the paper by Bezzina and Refalo [70], the cost of the printer (model Prusa i3) was estimated at EUR 800, while one injection molding machine was assumed to cost EUR 195,000, plus EUR 40,000 per mold. Finally, the discriminating factor is the nature of the products to be produced. AM, including FFF, is well suited to produce individual items or small batches of parts with complicated geometries and personalized features rather than large volumes of simple and identical objects because the production can easily be switched from one geometry to the next one without changing any tools [74]. The sweet spot below which AM technologies become (environmentally and financially) competitive over conventional ones should be assessed on a case-by-case basis. In the “cosmetic packaging” case study, it was estimated that FFF would be more financially viable than injection molding if 20,000 or fewer compacts were to be produced per year, where the trade-off point was still relatively high due to the considerable tooling costs for producing the injection molds [70]. Regardless of potential economies of scale, it is worth noting that AM enables the fabrication of complicated geometries that would be unfeasible with conventional processing methods [75]. Similarly, multi-component interlocking parts (“non-assembly” components) can be printed in a single job, which eliminates expensive and lengthy joining operations post-fabrication [76]. Although this point goes beyond the scope of the present review, it is also worth saying that AM may unlock additional advantages along the value chain. For example, AM parts can be produced at the point of use, and the avoided transportation reduces delivery costs, lead time, and GHG emissions [77]. However, FFF parts usually show anisotropic mechanical properties and lower resistance than their injection-molded counterparts [78].

In the comparison proposed by Bezzina and Refalo [70], the same material (ABS) was adopted for both manufacturing methods. However, different materials may be required to meet the same functional requirements with different manufacturing technologies. For example, Ponticelli et al. [79] questioned what would happen if the brackets used in the overhead lockers of commercial aircraft were produced by FFF with carbon fiber-reinforced polyether ether ketone (PEEK) instead of cast aluminum. The original bracket was reverse-engineered, and a structural analysis was conducted on the acquired 3D model in order to confirm the load-bearing capacity of the carbon-reinforced PEEK component. Notably, the FFF bracket met all the structural requirements while also affording a substantial weight reduction of nearly 50% with respect to the conventional cast aluminum counterpart. According to the estimates published by Ponticelli et al. [79], this weight reduction would translate into a massive reduction in the annual fuel consumption, corresponding to around 1740 tons/year per single aircraft on the Italian route Rome–Milan. Obviously, this would offer both financial advantages, leading to a saving of approximately 320,000 EUR/year, and environmental benefits, lowering the emissions by 550 tons/year of CO_2_ per aircraft.

The importance of adopting new advanced materials when comparing FFF to conventional manufacturing in load-bearing applications was also confirmed by the techno-eco-efficiency analysis developed by Jayawardane et al. [80], which demonstrated that technically feasible pump impellers could only be produced by FFF if glass fiber-reinforced Onyx (polyamide (PA) + short carbon fibers) was chosen as feedstock material. Electricity consumption was found to be the major contributor to the EI of FFF, with high greenhouse gas (GHG) emissions caused by the combustion of black coal and natural gas accounting for 84.5% of Western Australia’s electricity mix. Nonetheless, when compared to CNC machining for producing the same impeller, FFF reduced the total EI by nearly 96% thanks to the more efficient material’s usage and, most of all, thanks to the lower energy embodied in the manufacturing equipment.

Another point worth considering is that design principles are different for AM and for conventional fabrication systems because physical objects are built in different ways [81]. As a result, assuming that the part’s geometry will remain the same through the shift from conventional manufacturing to AM may be misleading when defining the functional unit in an LCA. For example, Top et al. [82] pointed out that, in order for a laser engraving machine (LEM) to be conveniently fabricated by FFF, snap-fit mechanisms should be introduced instead of fasteners to reduce the number of components. Meanwhile, as long as the infill degree does not affect the functionality of the LEM, this parameter can be as low as 30%. With the printer being used in low-power mode, the adoption of FFF with a redesigned LEM could afford a 60.45% reduction in material consumption and an 85.59% reduction in CO_2_ emissions with respect to conventional manufacturing [82].

AM processes, and FFF among them, are generally recognized as material-efficient but energy-intensive [83]. Nonetheless, the entity of the EI associated with energy demand actually depends on the nature of the electricity being used. This point was investigated by Kreiger and Pearce [84] in their analysis of the environmental benefits potentially associated with distributed manufacturing. According to Kreiger and Pearce [84], the adoption of FFF for distributed manufacturing affords two fundamental advantages over conventional “centralized” processing methods. Firstly, the ability to change the infill degree allows parts to be produced with minimal material use while still providing sufficient structural strength to meet the application requirements. Secondly, since objects can be produced where they are needed, the embodied energy of transportation can be significantly reduced. Nonetheless, conventional (centralized) manufacturing leads to lower embodied energy in manufacturing owing to economies of scale. Whether distributed manufacturing is environmentally convenient or not largely depends on the source of electricity. For example, when Naef building blocks (a toy originally designed by Kurt Naef in 1956 and historically carved from wood [85]) are manufactured by FFF, the CED when printing with PLA is always lower than conventional manufacturing if electricity is generated in situ using solar photovoltaic (PV) technology. However, if traditional electricity is used instead, the CED for FFF is lower than conventional manufacturing only if the infill degree is lower than 79%. This suggests that the energy source becomes critical whenever a high infill degree is required for a given application. For example, producing a water-tight waterspout by conventional manufacturing always results in lower CED values than FFF, unless distributed photovoltaic energy is implemented for printing PLA parts. Finally, what emerges from the LCA conducted by Kreiger and Pearce [84] is that distributed energy generation—for example, with photovoltaic arrays—bolsters the environmental benefits associated with distributed manufacturing, with the additional advantage that solar panels can also be arranged in remote areas that cannot be reached by the conventional grid.

The importance of developing and choosing more sustainable energy sources was also highlighted by Nagarajan and Haapala [86], who compared FFF to injection molding and direct metal laser sintering (DMLS) to conventional metalworking. As already observed by Kreiger and Pearce [84], Nagarajan and Haapala [86] noticed that the EI caused by energy consumption in the form of electricity could be reduced by using an electricity mix based on renewable sources instead of coal.

##### Other AM Technologies

As already seen for conventional manufacturing, the environmental performance of FFF, as compared to other AM methods, should also be assessed on a per-case basis.

Ulkir [87] compared the EIs associated with FFF to those of stereolithography (SLA) when printing a rack of plastic tubes for a chemical lab and reached the conclusion that 3D printing of a photocurable resin by SLA was the most environmentally friendly approach in the mid-point and end-point indicators, regardless of the material being printed by FFF (ABS, PLA, PETG). However, it should be noted that this conclusion was largely influenced by the assumption that depolymerization, as originally described in the literature for a highly specialized thermoset (namely, an acrylate–epoxy hybrid resin receiving a two-stage photocuring process [88]), would be adopted as the common procedure for recycling any SLA-printed parts.

In a combined economic and environmental analysis, Tagliaferri et al. [89] noted that nesting multiple objects in the same job splits energy and labor costs and helps recover material costs in AM. However, due to the raster-by-raster build-up mechanism, these benefits are less substantial in FFF than they are in other AM technologies like selective laser sintering (SLS) and multi-jet fusion (MJF). This also leads to a relatively low annual production capacity, which is consistent with the observations of Bezzina and Refalo [70]. According to the LCA results published by Tagliaferri et al. [89], the main potential EI could be attributed to the depletion of resources, particularly fossil fuels, for all AM technologies under examination. Quantitatively, the greatest potential impacts were originated by FFF, followed by SLS (with different values for different printers), and finally by MJF, which performed best due to the lowest energy consumption. In terms of feedstock, the EI of the materials used in FFF was much lower than that of the materials processed by other technologies, but filaments for FFF were also more expensive.

One of the main shortcomings of FFF as compared to conventional manufacturing methods and other 3D printing techniques is its poor surface quality. Reaching a smooth surface finish may be unnecessary for some applications. This is the case, for example, of the bracket for the overhead lockers mentioned above in the paper by Ponticelli et al. [79], whose functionality was unaffected by the relatively high surface roughness. However, the surface quality may be critical when tight tolerances are needed. For example, the FFF printed pump impellers investigated in the contribution by Jayawardane et al. [80] had a relatively poor pumping efficiency due to the raster- and layer-induced surface waviness. Likewise, achieving a smooth surface is crucial when the object must satisfy high aesthetic standards. An example is given by the compacts produced by FFF in the contribution by Bezzina and Refalo [70], which failed to meet the quality of their injection-molded counterparts. As a result, post-processing may be needed to improve the surface finish of FFF parts to the same quality level afforded by other manufacturing technologies, either conventional or additive, and this must be included in the LCA. For instance, Kwon et al. [90] calculated that initial post-processing is responsible for more than half of the EI of FFF when producing a NIST test artifact, which is a standardized benchmark to quantitatively evaluate the performance of an AM system [91]. The results published by Kwon et al. [90] are consistent with the remarks by Faludi et al. [71], who also observed that additional processing to enhance the surface quality of FFF parts could deeply affect the results of an LCA and amplify the EI of FFF.

Finally, another important consideration that stems from the prevailing role of energy consumption in the environmental damage caused by AM is that lightweight design—and, hence, material saving—by itself is not sufficient to make AM “environmentally sustainable”. As for FFF, Enemuoh et al. [92] estimated that, for each unit mass of printed material, energy consumption is mainly attributable to the printing process itself, while the embodied energy in the raw plastic pellets and the filament extrusion process just make a minor difference. This means that reducing the printing energy demand is the real challenge that must be solved in order to minimize the EI of FFF. This was also demonstrated by Mele et al. [93] for FFF-printed finger splints having a patient-specific shape. For comparative purposes, the splint was designed by means of generative design, topology optimization, and the use of lattice structures. A desktop FFF printer and an industrial-scale Arburg plastic freeforming (APF) system were then considered for fabricating the splint (where the Arburg freeformer is also a MEX printer like FFF, but using plastic granules instead of filaments [94]). A design of experiment (DOE) was planned to understand how the EI of the two printing methods was affected by the design strategy (including the “full”—i.e., not lightweighted—geometry of the splint with different infill degree values), by the orientation of the part on the base platform (which strongly influences the build time and, hence, the energy consumption), and by the number of parts being built simultaneously (one part per job vs. the maximum number of parts nestable on the base platform). APF systematically required more time for printing due to its droplet-by-droplet build strategy, which is inherently slower than the continuous deposition of matter underpinning FFF. Moreover, all the lightweighted design solutions required support structures, but the support material was less for FFF owing to its better bridging ability. As for the different design options, reducing the infill degree substantially shortened the time for printing the “full” splint. Generative design and topology optimization also resulted in a shorter printing time than the “full” splint with 100% infill degree since less material was needed, and hence, fewer movements were required. However, while this effect was noticeable for FFF, it was almost negligible for APF. Interestingly, the adoption of a lattice structure had the opposite effect on the printing time, which increased due to extensive contouring. Printing the splint at an angle of 45° or laying down on the build platform was disadvantageous due to the increased need for support. In accordance with these figures, all the EIs on “Human Health”, “Ecosystem Quality”, and “Resources Depletion” were higher for APF than for FFF. Additionally, the environmental burden of APF was worsened by the energy needed for heating the large base platform and by the adoption of disposable build plates. Notably, the contribution of material consumption to the EIs was almost negligible for both technologies. Conversely, most indicators were governed by the machine life cycle and by energy consumption, which were rather determined by the printing time. As a result, the design solution minimizing the printing time and not the material consumption emerged as the most environmentally sustainable option [93].

#### 5.2.2. What Is the Effect of the Printing Parameters on the EIs of FFF?

**Figure 9:**
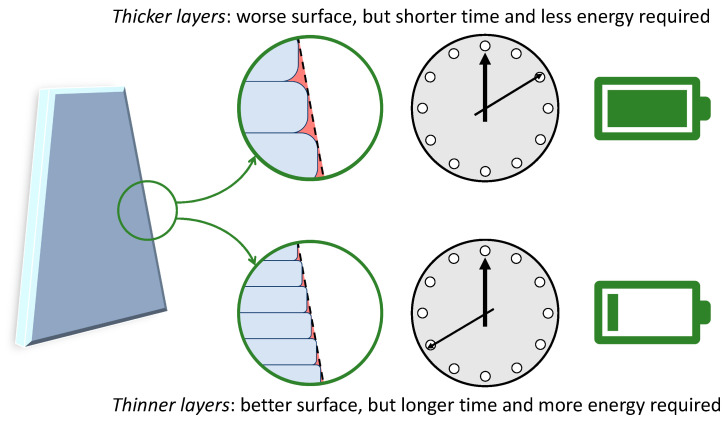
Stair-stepping effect, printing time, and energy consumption are associated with different values of the layer thickness in FFF.

For a given set of printing parameters, the power required for operating the printer is constant, and therefore, the energy consumption increases linearly with the duration of the printing job [16,93]. Consequently, all those variables that affect the printing time will ultimately influence the energy demand [89]. For example, increasing the layer thickness in FFF while keeping all other parameters unchanged reduces the number of layers required to complete the targeted geometry. Since the time need to deposit each layer does not depend on the layer thickness, the printing time, and hence the energy consumption, become lower. However, this generally comes at the expense of the surface finish because thicker layers worsen the stair-stepping effect, as exemplified in Figure 9. Post-processing may thus be required to rectify the surface properties, which would cause additional energy demand, as noted above. Meanwhile, as discussed in the literature, changing the layer thickness may have complicated consequences for the mechanical properties of the printed part [29]. Printing thinner layers requires a higher exit pressure from the nozzle, and this forces the new layer against the previous one, thus ameliorating the inter-layer adhesion [95]. However, thicker layers cool down more slowly. In this way, polymer chains can retain some mobility for a longer time, which helps heal the inter-raster interfaces. Meanwhile, since thermal gradients are reduced, thermal stresses also become less damaging with respect to parts with thicker layers [96].

A detailed analysis of the effect of different printing conditions on the EIs of FFF due to energy consumption was developed by Mecheter and Tarlochan [97]. The geometric complexity of the part to be printed had a minimal impact on the energy consumption when ABS and tough-PLA were used. However, the energy consumption slightly increased with higher geometric complexity when printing with PLA. For all printing materials, a substantial increase in energy consumption was associated with a reduced layer thickness. As previously explained, this is mainly due to the longer time needed to complete the part. Similarly, energy consumption increased with a higher infill degree [97]. The role of the infill degree in determining the EI of FFF is actually two-fold because parts with a higher infill degree require more feedstock material to be built, which is the main driver for resource depletion, and also need more time and, hence, more energy to be printed, which is the driver of impacts on human health and the environment [98,99]. Intuitively, as recommended by Kreiger and Pearce [84] and by Ma et al. [99], the infill degree should be carefully adjusted to meet the service requirements, as overperforming implies unnecessary environmental loads.

Interestingly, according to the results presented by Mecheter and Tarlochan [97], if the infill degree is assumed to be constant, the choice of the infill pattern does not affect energy consumption. This implies that the leading variable is the amount of material being deposited rather than its location, although long travels of the printhead may require more time and increase the energy demand with respect to short travels [97].

According to the results presented by Mecheter and Tarlochan [97], for a given printing material, the change in energy consumption is negligible when the printing temperature is varied within the range recommended by the filament’s manufacturer. However, as further discussed in the following section, the printing temperature of the feedstock material is indeed relevant because the lower the printing temperature, the lower the energy consumption.

Ultimately, Mecheter and Tarlochan [97] observed that a higher printing speed corresponds to a lower energy consumption, owing to the shorter time needed to complete the printing job. However, the calculations by Ma et al. [99] suggest that increasing the printing speed decreases the energy demand less than expected because the start-and-stop operations become more frequent, thus taking more energy from the stepper motor, and because the increased material flow through the liquefier requires faster heating.

Besides printing, additional energy may be consumed in FFF for (pre-)heating the base platform [99]. Working with a heated platform is often strongly recommended to mitigate thermal residual stresses, prevent the part from peeling off, and improve inter-raster and inter-layer healing [100]. Sometimes, the whole build chamber must be heated up to facilitate the printing of “thermally demanding” feedstocks, such as high-performance polymers [101]. Further to this, it is common practice in the industry to keep the printers on, even if they are not running, because this shortens the time required for ramping up and starting a new job. However, although the equipment is set to “stand-by” mode, energy consumption is only reduced but not totally interrupted [17]. As a result, the EI of FFF can be mitigated by printing multiple parts in the same job (“nesting”), because this decreases the electricity consumption allocated to each product during the stand-by and warm-up stages [92,102].

#### 5.2.3. What Is the Effect of the Feedstock Material on the EI of FFF?

According to Nagarajan and Haapala [86], the damage to resource availability associated with FFF is mainly due to fossil resource depletion (99%) and metal resource depletion (1%) due to polymer production and electricity consumption. In addition to the uptake of more sustainable energy sources (as discussed above), Nagarajan and Haapala [86] suggested that the shift to bio-based or recycled materials would also help reduce the resource depletion issue. This argumentation was also supported by the LCA conducted by Fico et al. [103] to compare the EIs associated with various PLA composites reinforced with olive wood waste. The production of PLA granules, followed by grinding and drying, was identified as the largest contributor to GWP and “Abiotic Depletion Potential—Fossil Fuel” for all materials. Due to the addition of wood, energy consumption for extruding and printing increased over neat PLA. However, as neat PLA was replaced by olive wood waste, which had lower embodied energy, the total impact decreased almost linearly, by 5.5% with 10 wt.% of wood and by 10% with 20 wt.% of wood.

PLA is widely acknowledged as a “green” feedstock material for FFF because it is bio-based and renewable [104]. For example, besides having a lower melting point and hence a lower printing temperature, PLA has a lower carbon footprint throughout its entire life cycle when compared to ABS [97]. However, PLA is sensitive to moisture uptake. Absorbed water molecules change the mechanical behavior and, hence, the printability of PLA because they act as plasticizers. Moreover, PLA is known to experience severe thermo-mechanical degradation upon processing due to the hydrolysis of the ester bond [105,106]. Successful printing largely relies, therefore, on pre-drying the filament, which is an energy-intensive operation. For this reason, in the contribution by Bay et al. [107], the EI of PLA was found to exceed that of recycled PP, although this result was likely conditioned by the different origins of the two polymers, with PLA being internationally produced and shipped and PP being locally recycled and managed. The environmental burden associated with pre-heating and drying is emphasized with materials like PA–matrix composites, which are even more labile than PLA [108].

If FFF parts are intended for load-bearing applications, the environmental friendliness of a feedstock material over another depends on the specific application and the operating loads the components must withstand. This was demonstrated, for example, by Bianchi et al. [109], who defined the functional unit of the LCA as “the production of a tensile specimen that exhibits a maximum strain equal to 2.55% when subjected to a tensile load of 3.1 kN and has a length of 170 mm” using either short glass-fiber reinforced or short carbon-fiber reinforced PA, termed GlassPA and CarbonPA, respectively. The reference geometry of the tensile samples complied with ASTM D3039. Due to the different stiffness of the two composite materials, the functional unit corresponded to a part of 18.21 g for GlassPA and 9.21 g for CarbonPA. Because of this disparity in weight, CarbonPA was characterized by the lowest EI for both endpoints under examination, namely GWP and CED, because the energy consumption depended linearly on the required printing time and, therefore, on the part’s weight. However, when the functional unit was redefined as “the production of a 3D printed sample that, during a flexural test, exhibits a maximum strain of 2% when subjected to a load of 556.8 N and has a length of 100 mm”, the mechanical performance of the two composites became comparable, and hence the part’s weight was also similar. As a result, the EI associated with GlassPA was lower than that of CarbonPA because the embodied GWP and CED of glass fibers are much lower than those of carbon fibers [109].

#### 5.2.4. Can Recycling Be the Way Forward and Reduce the EIs of FFF?

One of the main advantages of FFF over other polymer-based AM techniques, such as vat photopolymerization, is that thermoplastic materials can be reprocessed and recycled [12,110]. The treatment of recycling processes in LCA may require special attention since the analyst must decide how the impacts should be attributed to the life cycles involved or if a credit for the avoided virgin material should be considered instead [27]. Regardless of this methodological issue, as already discussed by Luo et al. [59] back in 1999, the EI of AM parts changes according to the way of managing end-of-life parts, with the best environmental performance (i.e., minimum EI) being associated with recycling, whereas the values for landfilling and for incineration were found to be quite similar.

As for FFF, Zhao et al. [111] observed that close-looped recycling, whereby recycled PLA parts are used instead of virgin polymer for producing printable filaments, and incineration, whereby electric power can be generated from recovered energy (heat), enable significant environmental savings as compared to landfilling, which only produces environmental burdens. Recycling can also be favorable for saving natural resources, especially water, because it avoids the substantial water consumption required for polymerizing virgin PLA. According to Zhao et al. [111], the main benefits of incineration are associated with the terrestrial acidification potential (TAP) and the ozone depletion potential (ODP) categories because the generation of electric power in China still largely relies on coal firing (which reconfirms the importance of the energy source being used for powering the manufacturing system, as discussed above). Among other impacts, landfilling is finally associated with the extensive release of persistent organic pollutants and particles [111].

As pointed out by Zhao et al. [111], besides preserving natural resources, recycling affords additional benefits. Firstly, recycling is more and more often necessary to meet the stringent environmental policies being enforced in many countries [112,113,114]. Additionally, recycling may be economically profitable as it only requires energy for shredding, drying, and extruding, which can be estimated at less than USD 1 for 1 kg of PLA, which is far less than the cost of virgin PLA pellets, currently sitting at about USD 18 per 1 kg of virgin PLA [111]. Likewise, printing spools made of recycled PETG may cost between 4 and 10 USD/kg, while a spool of virgin PETG is generally around 25 USD/kg [115]. Recycling also takes less time than synthesizing, granulating, and extruding virgin polymers, thus cutting down on time to market [111].

On account of the continuous advancement of composite materials as functional feedstock for FFF [116,117], increasing attention has been paid in the literature to their recycling. Meanwhile, FFF is emerging as a potential recycling route for polymer composites in various industries, including automotive, electronics, construction, sports, and leisure [12]. For example, FFF combined with mechanical recycling (crushing and sieving) can successfully valorize scrap glass fibers obtained from dismissed wind turbines [118,119,120]. The numbers in play are massive. According to the report released in February 2024 by AVK, just in Europe, the market for thermoplastic composites in 2023 had a total volume of 1423 kilotonnes (kt) [121]. FFF offers an additional option for recycling industrial composites that complements conventional approaches based on injection molding and other traditional manufacturing techniques. Owing to the relatively low investment costs required for purchasing the equipment (in principle, a simple recycling workflow would require a pelletizer, a desktop extruder, and a printer), FFF could contribute to the in-house recycling of small volumes of scraps and failed parts. Conversely, recycling through injection molding would likely require the involvement of a third (external) party specializing in this technology.

So far, relatively few contributions in the literature have applied LCA methodologies in order to assess the EIs specifically associated with the recycling of composites by FFF. Recycling composite materials and filaments may be challenging due to the coexistence of heterogeneous phases, and this may also lead to additional environmental burdens [12]. One of the main hurdles is the possible recovery of fibers from composite waste. In principle, after removing the thermoplastic matrix by either thermal (thermolysis) or chemical (solvolysis) means, fibers can be reused. However, the recovery process is likely to cause fibers to degrade, and the functionality loss governs the product’s lifetime. Chatzipanagiotou et al. [102] analyzed the EI of two different solvolysis processes for carbon fiber recovery, namely supercritical solvolysis and plasma-enhanced solvolysis. When compared to landfilling as an end-of-life treatment, both solvolysis methods led to negative EIs (i.e., environmental benefits) across most impact categories since the recovered carbon fibers could be credited as an avoided product. Interestingly, when compared to the synthesis of virgin carbon fibers as a secondary process yielding fibers, both solvolysis processes enabled significant environmental benefits in most impact categories, even assuming a 50% loss of functionality. In terms of the solvolysis process, supercritical solvolysis had the lowest EI across all impact categories. In its turn, plasma-enhanced solvolysis had a lower EI compared to conventional processes in all impact categories except for ionizing radiation, where the ionizing radiation category was affected by Radon-222 being originated from the nuclear power production included in the Ecoinvent market group for medium-voltage electricity within the geography “Europe without Switzerland”, as per the background database used by Chatzipanagiotou et al. [102]. Consistent with the reduced EI associated with recovered fibers as compared to virgin ones, products receiving recovered fibers had a lower EI than the baseline product, even if the fibers lost 50% of their functionality, with the main exception being the ionizing radiation category [102].

Finally, additional research is still needed to facilitate the recycling of composite materials through FFF [12]. Composites should be made “sustainable by design”, for example, by minimizing the compositional difference between polymer matrix and reinforcement [122]. In this regard, self-reinforced polymer composites could be a possibility because the matrix and the reinforcement have an analogous chemical makeup but different structures [123]. However, the melting temperature would also be similar for both constituent phases, and finding a proper processability window would be difficult on account of the two heating-and-melting cycles associated with filament extrusion and FFF printing [124]. Certainly, as mentioned above, the filler loading should match the service requirements with as much accuracy as possible because overfilling and oversizing always imply unnecessary environmental burdens [99].

## 6. Conclusions

Fused filament fabrication (FFF, or fused deposition modeling, FDM) is currently the most widespread material extrusion (MEX) additive manufacturing (AM) method, as well as the commonest plastic-based 3D printing tool. The efficient feedstock material usage, the reduced need for transportation through distributed manufacturing, and the recyclability of thermoplastic-based filaments contribute to the general perception of FFF as a “green” fabrication technology. Nonetheless, the sustainability of FFF should be more carefully analyzed due to its capillary diffusion for industrial and recreational purposes, which may lead to environmental and societal challenges. A systematic review of the literature revealed that life cycle assessment (LCA) is being applied more and more broadly to determine the environmental impact (EI) of FFF as compared to conventional manufacturing methods and other 3D printing technologies and also to explore potential mitigation strategies based on the development of new feedstocks and the adoption of recycling.

Although the results of an LCA are always case-specific, it is clear that one of the main environmental drawbacks of FFF is the substantial energy consumption, as is commonly seen for most AM methods. As a result, FFF is often environmentally disadvantageous with respect to faster and less energy-intensive technologies like injection molding, especially when large batches of identical parts are to be produced. However, whether FFF is environmentally more convenient than other technologies or not largely depends on the source of electricity being used. The shift to renewable sources, such as photovoltaic generation, is expected to play a key role in promoting the sustainability of FFF.

Meanwhile, the EI of FFF can be reduced by cutting down the printing time, which is directly correlated to energy consumption. For example, nesting multiple items in a single printing job may be beneficial for lowering the embodied energy per part. The effect of the layer height is less obvious since increasing the layer height shortens the printing time but worsens the surface quality to the point that post-processing may become necessary to meet specific functional or aesthetic requirements. 

Since the consequences of feedstock material usage are often negligible with respect to those of high energy consumption, changing from traditional geometries to lightweighted ones (for example, topologically optimized lattices) may not be sufficient to lessen the EI of FFF. However, since FFF is capable of producing hollow parts, a parameter of paramount importance is the so-called infill degree, which defines the amount of material actually used to print the interior of a part. The infill degree can be adjusted to satisfy the structural requirements for a given application while enabling substantial savings in terms of material and printing time/energy.

Finally, choosing feedstock materials that are bio-based or that can be recycled can help reduce the depletion of natural resources, including fossil fuels and water. However, research is still needed to overcome some material-related issues, such as the pre-drying step of moisture-sensitive filaments, and to remediate the functionality loss generally caused by recycling.

## Data Availability

Data will be made available upon request.

## Supplementary Materials

The following supporting information can be downloaded at: https://www.mdpi.com/article/10.3390/polym16141986/s1, Table S1: summary of primary sources.
