# Supplementary material for: Environmental Impact of Fused Filament Fabrication: What Is Known from Life Cycle Assessment?
Source: Polymers (Basel). 2024 Jul 11;16(14):1986. doi: 10.3390/polym16141986 (PMC11281121; doi:10.3390/polym16141986)
Supplement: Supplementary file 1 [file polymers-16-01986-s001.zip › polymers-3072226-supplementary.pdf]

**Bibliographic information**							**LCA**								
**Bibliographic record**				**Geography**			**Scope of the LCA**	**What was analysed**			**How it was analysed**			**Results**	
Authors	***Title***	***Journal/source***	***Year***	***First author's country***	***Corresponding author's country***	***No. of countries***	***Reason***	***Feedstock***	***Boundary***	***Functional unit***	***Database inventory***	***Software used***	***Impact assessment method***	***Main findings***	***Limitations/Notes reported in the paper***
Comparison between FFF and conventional technologies and/or other AM methods															
Bezzina C.M., Refalo P.	Fused filament fabrication and injection moulding of plastic packaging: An environmental and financial comparative assessment	Machines	2023	Malta	Malta	1	Comparison of FFF to injection moulding (IM), associated with cost analysis	ABS	1.5 million compacts (= cosmetic plastic packaging) yearly for 12 years (where 12 years is the expected lifetime of the IM system, against 5 for FFF printers)	Raw material extraction, processing, manufacturing (not assembly, transportation, use, and end-of-life stages, as common to both FFF and IM objects)	Ecoinvent v. 3<br>Ansys GRANTA Edupack for ABS production<br>Literature data for extrusion for FFF	SimaPro 8.4	ReCiPe 2016, Midpoint and Endpoint (human health and ecosystem endpoints)<br>Sensitivity analysis to test if the assumptions of machinery production are sensible	FFF produced a five times greater environmental impact on both endpoints, with printing energy consumption generating 80% of the impact (The printer consumption to produce one compact was twenty times greater due to the 5-hour FFF cycle time compared to 7s);<br>The human health EI is much greater than the ecosystem EI for both technologies.<br>Energy consumption is case-specific. This leads to different results in environmental assessments comparing 3D printing with conventional technologies such as IM.<br>3D printing could still potentially have a smaller overall carbon footprint when considering the potential to reduce the material usage and weight of the product. With process parameter optimisation, the energy consumption could be decreased.<br>Meanwhile, FFF maximises material utilisation (greater impacts generated by IM raw material production when compared to filament production).<br>The net present value (NPV) of FFF was seventeen times higher than that of IM.<br>The greatest cost for FFF was the material cost, which is twelve times greater for filaments compared to the ABS granule cost for IM;<br>Labour was the second greatest cost for FFF; machinery and energy consumption were also more expensive. However, at 20,000 or fewer compacts yearly, FFF would be more financially feasible than IM due to the tooling costs required.<br>The quality of packages produced using IM were superior overall.<br>In conclusion, FFF is more expensive and environmentally impactful than IM.	The FFF printer production and printing process were not found in the Ecoinvent database and had to be manually created using assumptions; the same approach was followed for the IM system for comparison (basically, it was assumed that a printer is 100% aluminium, and an IM system 100% steel)
Enemuoh E.U., Menta V.G., Abutunis A., O'brien S., Kaya L.I., Rapinac J.	Energy and eco-impact evaluation of fused deposition modeling and injection molding of polylactic acid	Sustainability	2021	U.S.A. (MN)	U.S.A. (MN)	1	Comparison of FFF to injection moulding	PLA	Cradle to gate	1 kg of PLA for embodied energy in PLA feedstock<br>ASTM D638 Type IV dogbone for "forming energy"	Literature data from ANSYS Granta	Analytical equations for estimating energy consumption		For 1 kg of printed material, power consumption is mainly attributable to the printing process itself, while embodied energy in PLA pellets and extrusion process make a minor quote.<br>The power consumption during FFF of one dog bone-shaped sample (6.53 g) was measured at 0.331 kWh, but the total power consumption should also account for the extrusion energy.<br>The total power consumption during injection moulding to produce the same dog bone was measured at 0.474 kWh.<br>Nesting multiple parts in one job enables energy savings.	Although formally presented as "LCA", the paper does not adhere to international standards. The manuscript develops an analytical model for estimating energy consumption and CO_2 emissions in FFF and in injection moulding.
Faludi J., Bayley C., Bhogal S., Iribarne M.	Comparing environmental impacts of additive manufacturing vs traditional machining via life-cycle assessment	Rapid Prototyping Journal	2015	U.S.A. (CA)	U.S.A. (CA)	1	Comparison of FFF to polyjet/inkjet to CNC machining	ABSplus (modelled as ABS) + SR-30 for FFF<br>ABS-like (fullcure 720) + SUP705 for inkjet<br>ABS blocks for CNC	Cradle to grave (including embodied impacts, transportation, energy used during manufacturing, energy used while idling and in standby, material used in final parts, waste material generated, cutting fluid for CNC, and disposal	One "job" comprising the manufacturing of two different parts in plastic – one with complex curvature, as people often make by AM, and the other with simple planes and holes, as people often make with traditional milling machines		SimaPro	ReCiPe, Endpoints, Hierarchist; IMPACT 2002+ methodologies; Midpoints normalised and weighed to a single score impact	The sustainability of AM vs CNC machining depends primarily on the per cent utilization of each machine.<br>At both high and low utilization, the FFF machine had the lowest ecological impacts per part. The inkjet machine sometimes performed better and sometimes worse than CNC, depending on idle time/energy and on process parameters.<br>For both AM printers, electricity use is always the dominant impact, but for CNC at maximum utilization, material waste became dominant, and cutting fluid was roughly on par with electricity use.<br>Although the major sources of impacts change from 3D printing to CNC milling, the distribution of impacts by type of ecological damage remain fairly constant – fossil fuel depletion and climate change remain dominant, with notable contributions from particulates and human toxicity. This is because plastic production (the largest impact for CNC) and the average US electricity production (the largest impact for FFF and inkjet) are both fossil fuel industries.<br>Changing quality levels might change environmental impacts.	The LCA did not include post-processing that might bring the surface finish of FFF parts up to the quality of inkjet or CNC parts;<br>The LCA was limited to environmental impacts; societal impacts were only considered qualitatively;<br>Different polymers had to be used for different AM technologies; "ABS-like" is quite different from ABS, thus requiring a sensitivity analysis<br>Estimations of mass for FFF had to be increased by 20% with dummy inventory items, but in two of the three scenarios studied, the FFF machine's embodied impacts were insignificant<br>Cutting fluid is not required for CNC milling of plastics, but included for comprehensiveness
Garcia F.L., Nunes A.O., Martins M.G., Belli M.C., Saavedra Y.M.B., Silva D.A.L., Moris V.A.D.S.	Comparative LCA of conventional manufacturing vs. additive manufacturing: the case of injection moulding for recycled polymers	International Journal of Sustainable Engineering	2021	Brazil	Brazil	1	Comparison of FFF to injection moulding, associated with analysis of quality requirements (tensile strength)	ABS recycled from Waste Equipment Electric Electronics (WEEE)	Cradle to gate	1-sample production of a specimen with the same shape and dimensions as described by the ASTM D638, in batches of 7 for FFF and IM, and batches of 14, 50 and 100 for IM	Unit Process Life Cycle Inventory (UPLCI) methodology by Kellens et al.;<br>inventory data from literature (analysisng the recycled ABS in use)	SimaPro 8.4.1.4	ReCiPe 2016, Hierarchist, Midpoint v1.00;<br>Global Warming Potential (GWP) as per Impact 2002+ v2.14 and ReCiPe 2016;<br>Cumulative energy demand (CED) v1.09.<br>Sensitivity analysis for different infill degrees	GWP decreases for increasing batch size in IM due to reduced amount of energy required per part, but becomes nearly constant for 50+ part batches.<br>GWP of FFF decreases linearly over decreasing infill degree (less material used).<br>For 7 and 14 part batches, GWI is lower for FFF than IM, regardless of the infill degree.<br>Conversely, for 50 and 100 part batches, GWI is lower for IM than FFF, regardless of infill degree.<br>Main contributions to GWP of FFF are material used, base platform heating, and printing operations.<br>CED decreases for increasing batch size in IM due to reduced "share" of injector heating impact per part, but becomes nearly constant for 50+ part batches.<br>CED of FFF decreases over decreasing infill degree.<br>For 7 and 14 part batches, GWI is lower for FFF regardless of the infill degree.<br>Conversely, for 50 and 100 part batches, GWI is lower for IM than FFF, regardless of the infill degree.<br>Main contributions to CED of FFF are base platform heating and printing operations.<br>However, for tensile strength:<br>- Tensile strength of FFF parts decreases with increasing infill degree;<br>- even for 100% infill, the tensile strength is higher for IM than FFF (by 19%)<br>Mechanical eco-efficiency can be defined as UTS/GWP - this (positive) indicator increases with increasing infill degree, signifying that the increase in mechanical strength offsets the increase in GWP.	ABS is extruded and pelletised before injection moulding; since extrusion is common for both fabrication processes, it is not included in LCA.<br>Gas emissions were excluded from the system boundaries due to lack of data.<br>Base platform heating accounts for most GWP and CED in FFF due to the fixed heating time, irrespective of the initial temperature.<br>Rhe amount of material wasted in the ducts represents about 33% of the mass of the injected part as a result of the mould design.
Jayawardane H., Davies I.J., Leadbeater G., John M., Biswas W.K.	'Techno-eco-efficiency' performance of 3D printed impellers: An application of life cycle assessment	International Journal of Sustainable Manufacturing	2021	Australia (WA)	Australia (WA)	1	LCA combined with characterisation and other analysis tools in a 'techno-eco-efficiency' framework for impellers	Onyx<br>Glass fibre-reinforced Onyx	Conception to use (design, material processing, manufacture, use of the impeller, and transportation between stages)	Impeller delivering fluid over its useful life		SimaPro 8.4	Endpoints EIs:<br>Eco-toxicity EIs calculated based on individual values of human, terrestrial, freshwater, and marine toxicity.<br>Five EIs calculated using Australian indicator set with embodied energy V2.01<br>Two EIs calculated following EPD (2013) V1.02<br>Two EIs using ILCD 2011 Midpoint + V1.08 method<br>Remaining EIs using CML-IA baseline V3.03 as the Australian indicator set does not allow the calculation of these impacts.<br>Single score of EI:<br>EIs normalised and compounded through weights based on local survey with stakeholders from academia, industry and	The same goal, scope and life cycle inventory (LCI) are used for (environmental) life cycle impact assessment (LCIA) and for life cycle costing (LCC, inc. labour). Environmental impacts (EIs) and LCC results are normalised and plotted as eco-efficiency (EE) portfolio. If the product in unsatisfactory, cause-diagnosis and improvement strategies are applied iteratively. Feasibility of the techno-eco-efficiency framework is demonstrated for a semi-open pump impeller.<br>Only the glass fibre-reinforced impeller was technically feasible.<br>The FFF impeller had lower EI values compared to the CNC impeller.<br>Electricity consumption was found to be the major contributor for the FFF impeller, with high GHG emissions due to combustion of black coal and natural gas accounting for 84.5% of Western Australia's mix.<br>For the CNC benchmarks, EIs came from the production of the CNC machine itself.<br>The FFF impeller showed 82.5% lower production cost compared to the CNC impeller due to lower capital costs (75.2%), lower replacement costs (62.2%) and lower manufacturing costs (83.6%). FFF has much lower labour cost during manufacturing (no operator required during printing). Fibre-reinforced Onyx is more expensive than neat PA6, but less material waste. However, the operating cost of FFF impeller is higher due to lower pumping performance.<br>The total EI of the FFF impeller was found to be 96% lower than the CNC impeller.<br>Both impellers exhibited positive EE scores. However, the FFF impeller F exhibited comparatively higher EE.	Dimensions of impeller not given, but kept constant across FFF parts and CNC benchmark.<br>Impeller model based on conventional product in the market, thus benefits of topology optimisation to 3D printing were not explored.<br>Failure rate of 3D printers in mass manufacturing compared to mass manufacturing of CNC machined parts was not considered.<br>The end-of-life stage of the pump impeller was not considered in the scope of the LCA (and LCC) in order to focus on the feasibility assessment of 3D printed products.<br>Single score environmental impact (obtained by normalising, weighting, and aggregating endpoint environmental impacts) entailed uncertainties (and geographical assumptions) while presenting a simple method for comparative assertions

Kreiger M., Pearce J.M.	Environmental life cycle analysis of distributed three-dimensional printing and conventional manufacturing of polymer products	ACS Sustainable Chemistry and Engineering	2013	U.S.A. (MI)	U.S.A. (MI)	1	Comparison of distributed AM (FFF) to conventional manufacturing overseas with shipping PLUS quantification of distributed electricity generation system using solar photovoltaic (PV) technology	PLA<br>ABS	1:1, and input by kilograms required to create each individual product:<br>* Naef building block (0, 5, 10, 25, and 100% fill with PLA, and 25% with ABS)<br>* Water spout (100% infill degree as water-tight)<br>* Juicer (15% infill degree)	Cradle to gate (from raw material extraction to the product exiting the factory gate, with the gate located in the U.S.A. and hence including shipping to U.S.A. if overseas; packaging not included)	EcoInvent v. 2.0	SimaPro 7.2	Cumulative energy demand (CED 1.07);<br>IPCC GWP 2007 100a (global warming potential over 100 years in kg CO_2 equivalent emissions)	Potential advantages of distributed manufacturing through FFF (RepRap):<br>- the ability to change fill degree allows more complicated shapes to be produced with structural integrity while minimizing material use<br>- the potential reduction in embodied energy of transportation made available by distributed manufacturing<br>Potential advantages of conventional (centralised) manufacturing, among others:<br>- lower embodied energy during manufacturing of a given product because of scale<br>* Blocks: The cumulative energy demand (CED) depends linearly on the fill degree. If photovoltaic (PV) sourced, the CED of FFF with PLA is always lower than conventional manufacturing. If traditional electricity is used, the CED for FFF is lower than conventional manufacturing only if the fill degree is lower than 79%. The distributed manufacturing cases have the lowest emission values compared to traditional manufacturing for all cases, except for distributed without PV for 100%.<br>* Water spout: Because of the 100% fill degree, conventional manufacturing has lower CED values for all cases except distributed PLA + PV. The emissions were reduced using distributed manufacturing PLA + PV and ABS + PV.<br>* Juicer: energy consumption is always minimised with FFF; emissions are also lower, except for the ABS juicer without PV (build platform at high T for long).<br>Solar PV array is amenable to distributed energy generation, reduces emissions, and can be deployed in remote communities without access to the conventional grid.<br>Infill degree must be adjusted to avoid overperforming.	U.S. eco-profiles were used when available; otherwise, European eco-profiles were used.<br>Post processing was not accounted for, which implies underestimating cumulative energy demand and emissions.<br>Overseas shipping distance is an underestimate due to taking a straight-line trip across the ocean.<br>Model simplifications were made, e.g. packaging not included.<br>PLA and ABS were used as an example, but may not be the ideal materials for these products or may require additional coatings to make them food-grade or child-safe.<br>A more detailed study needs to evaluate the embodied energy in equipment (both for conventional and distributed)
Krishna L.S.R., Srikanth P.J.	Evaluation of environmental impact of additive and subtractive manufacturing processes for sustainable manufacturing	Materials Today: Proceedings	2021	India		1	Comparison of FFF to CNC machining for producing aerospace parts			1 bevel gear<br>1 PCB support		SimaPro		AM: highly efficient material usage, high energy consumption (depending on part's "complexity", actually size)	
Kwon J.Y., Kim N., Ma J.	Environmental sustainability evaluation of additive manufacturing using the NIST test artifact	Journal of Mechanical Science and Technology	2020	Republic of Korea	Republic of Korea	1	Comparison of FFF, material jetting (polyJet), and PBF/SLS with small and large printbed size	ABS + soluble support for FFF<br>ABS + soluble support for PJ<br>PA 12 for both PBF/SLS	1 NIST artifact<br>200 NIST artifacts	Cradle to gate (manufacturing one part and post-processing (support removal inc.), no transportation)	Ecoinvent<br>Energy consumption for material production from literature	SimaPro 8.5	Eco-indicator 99 (11 parameters)	Scope: determining which factor is most influential among: 'object material', 'support material', 'additional material', 'power for printing', and 'power for support removal' which are the inputs of the life cycle inventory.<br>Small-bed size PBF/SLS (LSb) is the least harmful to the environment for low- volume-production, while large-bed size PBF/SLS (LSa) is the least harmful for high-volume-production, while polyjet (PJ) has the largest impact amongst the four.<br>Under low-volume production, the dominant element is 'power for printing' for PJ, LSa and LSb, while it is 'additional material' for FFF. Under high-volume production, the dominant element becomes 'additional material' for LSa, and 'object material' for PJ, LSb, and FFF. Initial post-processing is responsible for more than half of the environmental impact of FFF for one part (consistent with the remarks by Faludi et al. regarding EDM).<br>The most influential element of each varies according to the part orientation.	The difference between the current and previous studies may have been induced by<br>- the different layer thickness<br>- the fact parts here were printed one at a time<br>- the fast advancement of AM technology, along the line of the suggestion of Morrow et al. [J. Clean. Prod., 15 (2007) 932-943] who pointed out that the rapid growth of AM technology requires a re-evaluation of the environmental burden of the processes of newly developed machines.
Luo Y., Ji Z., Leu M.C., Caudill R.	Environmental performance analysis of solid freeform fabrication processes	IEEE International Symposium on Electronics and the Environment (1999)	1999	U.S.A. (NJ)		1	Comparison of the EI of "solid freedom fabrication" methods including FFF, SLS, SLA		1 kg of each material processed	Cradle to grave (material preparation, build, post-process, use, and disposal)	Eco-indicator, collected and calculated by PRé Consultants of Netherlands		Eight EIs defined as Material Extraction (ME), Material Production (MP), Energy Consumption (EC), Residue (RS), Material Toxicity (MT), Landfill (LF), Waste Processing (WP0 and recycling (RC)	Tabulated results show that the EIs largely depend on the printer in use, because of the different energy consumption.<br>For a given printer, the EIs change according to the way of disposing of end-of-life parts, and increase in the order recycling --> landfilling --> incineration, where the values for the last two are quite similar.	Published before LCA standards were issued.
Mele M., Pisaneschi G., Ciotti M., Campana G., Zucchelli A., Fiorini M.	Environmental drawbacks of lightweight design algorithms in material extrusion additive manufacturing: A case study	Journal of the Brazilian Society of Mechanical Sciences and Engineering	2023	Italy	Italy	1	LCA to confute the assumption that lightweight design reduces the environmental impact of FFF and Arburg Plastic Freeforming (APF)	PA6 with PVA (washable) supports	Cradle to gate	Patient-specific finger splint	Ecoinvent v. 3<br>Plus Product Environmental Footprints (PEF) for PVA		ReCiPe 2016, ref. [42] Campana et al. (2017), Endpoints	The contribution of material consumption to EIs is marginal, which implies that lightweight design itself is not sufficient to reduce the EIs.<br>* the EIs of complicated lightweight designs, such as lattices, may be higher than those of the original (fully solid) part in spite of material savings;<br>* EIs are mainly governed by machine life cycle and energy consumption, both of which are allocated on the basis of the printing time;<br>* all EIs increase almost linearly with the building time per part.	The geometry of the initial part and its application strongly affect the quantitative results of the LCA.<br>Any comparison between different AM technologies is strongly influenced by the different nature of the equipment (industrial printer for APF, with heating chamber and disposable build plate; desktop printer for FFF).<br>Results cannot be extended to other AM that rely on completely different build mechanisms.
Nagarajan H.P.N., Haapala K.R.	Characterizing the influence of resource-energy-exergy factors on the environmental performance of additive manufacturing systems	Journal of Manufacturing Systems	2018	U.S.A. (OR)	U.S.A. (OR)	1	Comparison of FFF to injection moulding, and DMLS to conventional metalworking	ASA<br>(Iron)	Cradle to gate (plus recycling?)	1 kg of each material (Actually, 1 kg of finished product, as one-kilogram part)	Ecoinvent v. 3.0	SimaPro 8.1	Cumulative exergy demand (CExD)<br>ReCiPe 2008, Egalitarian and Individualist and Hierarchist, Endpoints;<br>IPCC 2013 (global warming potential, GWP, on time horizons of 20, 100, and 500 years)	A generalized exergy analysis framework adopted from Gutowski et al. [Environ Sci Technol 2009;43:1584–90] was applied to DMLS and to FFF.<br>Lost exergy to the environment is 89.83% of the total input exergy of the DMLS system, while lost exergy is 94.17% of system inputs for FFF.<br>The cumulative exergy demand (CExD) of DMLS is almost thirty times more than traditional metalworking; the CExD of FFF is almost ten times more than injection moulding.<br>Endpoints: The damage to human health caused by both AM systems is predicted to dominate the other damage types (i.e., ecosystem quality and resource availability). The impacts of energy used in the form of electricity dominate the material-related impacts of the system if energy is mainly produced from coal, but the impacts of energy can be reduced by using a different electricity mix from renewable sources (demonstrated for DMLS).<br>For FFF, the damage to resource availability was attributed to fossil resource depletion (99%) and metal resource depletion (1%), due to polymer production and electrical energy use. The shift to bio-based or recycled polymers may help reduce it.	Although the boundary is defined as cradle to gate, iron and ASA are assumed to be recycled.<br>Unused iron powder is considered "waste", but then assumed as fully recycled.<br>While the functional unit is defined as "1 kg of each material", the term of comparison for DMLS is "traditional metalworking to produce a one-kilogram product made of iron (Fe).", and for FFF is "injection molding to produce a one-kilogram product made of ASA polymer material".
Ponticelli G.S., Venettacci S., Tagliaferri F., Guarino S.	Fused deposition modelling for aeronautics: Techno-economic and environmental assessment for overhead locker supports replacement	International Journal of Advanced Manufacturing Technology	2023	Italy	Italy	2	LCA and techno-economic assessment for replacing cast aluminium with reinforced PEEK by FFF	Carbon-reinforced PEEK (comp. 7075AA)	Cradle to grave (manufacturing, operating, and dismissing)	Production batch of 352 brackets to be installed in a medium-sized commercial aircraft, operating on the Rome-Milan route for 25 years of life	Literature data,<br>technical data sheets,<br>database	SimaPro 7.1	CML 2001 baseline impact method,<br>Impact scores normalized with reference factors for comparison	Workflow: reverse engineering of cast aluminium part; structural analysis by finite element method (FEM); proof of concept by printing; cost assessment; LCA.<br>Costs included: production; fuel; disposal - over an expected operating life of 25 years, with an average of 352 brackets in a medium-sized aircraft (Airbus 320).<br>End-of-life brackets are disposed of in a sanitary landfill, without disassembling the basic materials, or without the possibility of recycling them.<br>Both materials comply with the Von Mises yield criterion; the av. total displacement increases from 0.0145 mm to 0.1265 mm, but is acceptable.<br>Composite parts are rough, but there are no specific surface finish requirements; worst error on dimensional accuracy is 1.33% on hole's diameter.<br>* Costs: Production costs are higher for FFF due to the raw material's cost about 100 times higher, but costs can be reduced by nesting several parts/job (less labour for printer's set-up). FFF ensures a significant reduction in fuel consumption. Disposal cost increases. Finally, the economic benefit due to the fuel consumption reduction prevails on the increase in production and disposal costs, with an expected overall saving of 7.71×10^6 € over 25 years.<br>* LCA: For both materials, the totality of the impact is due to the operating stage, with negligible contributions from production and disposal.<br>EIs are higher for FFF in the disposal stage (composite not recyclable, as opposed to aluminium), but lower for production (except for ODP) and much lower for operation due to fuel savings.	Data refers to an Italian context (flight route, electricity, and dismissing/disposal procedures).<br>Due to lack of data, the consumption of kerosene was replaced by diesel.<br>Since the chosen "heat diesel" item takes into account the upper heating value for the fuel, the required energy was increased by about 6.3% in order to respect the energy balance.
Reddy R.D.P., Elgazzar H., Sharma V.	Investigations of personalized and sustainable approach of oral drug delivery systems through additive manufacturing	Rapid Prototyping Journal	2022	India	India	2	Comparison of FFF to conventional tableting	PVA impregnated with ascorbic acid (vitamin C)	100 tablet units, each tablet containing 5 mg of ascorbic acid	Cradle to gate<br>(excluding the transport and storage logistics during the production of tablets)	Ecoinvent v. 3.0<br>Syntegon Technology GmbH, Germany, for energy consumption in conventional tableting	SimaPro 9.1.0.8	ReCiPe 2016, Midpoint v. 1.03, normalized to a single impact score	FFF had minimal adverse impact when compared with the conventional method, except for five categories.<br>CO_2 emissions (global warming), human toxicity, water consumption and impact on fossil and mineral resources were low for FFF as the procedure consumed low electricity energy owing to fewer processes involved, used mostly safe chemicals and had minimal wastage due to near-net-shape fabrication property.<br>After normalisation, there was a drastic decrease by 91.3% and 84.2% in the case of terrestrial ecotoxicity and human carcinogenic toxicity for FFF.	The high carcinogenic susceptibility of conventional tableting was mainly due to some processing chemicals used for sucrose and dextrose, which were not included in the formulation of the FFF tablets.

Tagliaferri V., Trovalusci F., Guarino S., Venettacci S.	Environmental and economic analysis of FDM, SLS and MJF additive manufacturing technologies	Materials	2019	Italy	Italy	1	Comparison of FFF, SLS, MJF	PA12	Cradle to grave, but not usage (transportation?) (i.e., cradle to gate plus disposal) including powder recycling	4-unit batch of component 1 (flange-like, 100,700 mm^3)	SimaPro software database	SimaPro 7.1	Percentage impacts according to Eco-Indicator 99 method (Pré Consultans BV) ---> cumulative impact (or single score) determined after normalising and weighting	Cost analysis:<br>- The cost of feedstock (per unit mass) is higher for FFF than other techniques;<br>- Annual production capacity is low for FFF due to the inability of building multiple objects simultaneously;<br>- Depending on the volume of the working chamber, nesting multiple objects in the same job splits energy and labour costs, and helps recover material costs;<br>LCA:<br>- The greatest potential impact is related to the consumption and depletion of resources, particularly fossil fuels, for all technologies;<br>- The greatest impact potentials are associated with FFF, followed by the two SLS printers, and finally MJF;<br>- MJF performs best due to the lowest energy consumption;<br>- The impact of raw materials used in FFF is much lower than other technologies.	Background data for PA6 instead of PA12;<br>Energy consumption approximated as the product of build time by nominal power usage.
Top N., Sahin I., Mangla S.K., Sezer M.D., Kazancoglu Y.	Towards sustainable production for transition to additive manufacturing: a case study in the manufacturing industry	International Journal of Production Research	2023	Turkey	India	2	Comparison of AM (FFF) to conventional manufacturing for producing a laser engraving machine	PLA	Cradle to gate	1 laser engraving machine	ECO-it database<br>Eco-indicator 99 application	ECO-it software	CO_2 emissions<br>Total indicator value	The LEM must be redesigned for taking advantage of FFF (for example, introducing snap-fit mechanisms instead of fasteners for reducing the number of components)<br>In order to reduce printing time and energy consumption:<br>- printer is used in low power mode<br>- since the infill degree does not affect the functionality of the LEM, infill density parameter is chosen as 30%.<br>The shift to FFF with a redesigned LEM leads to a 60.45% reduction in material consumption and 85.59% reduction in CO_2 emissions.	Lack of data required for a comprehensive LCA.
Ulkir O.	Energy-consumption-based life cycle assessment of additive-manufactured product with different types of materials	Polymers	2023	Turkey	Turkey	1	Comparison of FFF with different filaments and SLA	ABS<br>PLA<br>PETG<br>UV resin	Cradle to cradle, but not transportation and usage	A plastic test tube shelf consisting of three frames and two borders	Umberto Ecoinvent v. 3.1 database	Umberto NXT	ReCiPe, midpoints and endpoints	UV resin was the most environmentally friendly material in the midpoint and endpoint indicators.<br>ABS exhibited bad results on many indicators and was the least environmentally friendly.	Depolymerization as described in a single literature paper for a specialised thermoset is assumed as the common procedure for recycling any SLA printed parts, which is not the case for common thermosets.<br>There appears to be a mix-up of energy required for printing and energy required for recycling.<br>The same equations for estimating the energy consumption are applied to FFF and SLA, although SLA is not based on thermal processing.<br>It is assumed that the energy required for extrusion is proportional to specific heat and "temperature increase", with the proportionality constant being the same for all polymers (including UV resin).
Effect of printing parameters															
Campana G., Mele M., Ciotti M., Rocchi A.	Environmental impacts of self-replicating three-dimensional printers	Sustainable Materials and Technologies	2021	Italy	Italy	1	Assessment of the EI of a desktop FFF printer and its replicas	PETG	Cradle to gate; recursive algorithm for self-replication	Mass of the part; mass of the supports; printing time for the 35 PETG self-replicated kit	Ecoinvent v. 3.6		ReCiPe v.1.04, Hierarchist, Endpoints	The building time is found to be the driver of impacts on human health and the environment, while the part mass governs the resource depletion.<br>The impact indicators increase moving from the original to the replicated machine. However, the resource depletion indicator becomes lower, owing to the weight saving associated with low infill degree.<br>Over generations, all the indicators converge to a constant value regardless of initial hypotheses.	Due to the lack of precise information about the machine production phase, the LCA is not expected to provide accurate quantification of the environmental impacts.
Ma H., Zhang Y., Jiao Z., Yang W., He X., Xie G., Li H.	Comprehensive assessment of the environmental impact of fused filament fabrication products produced under various performance requirements	Journal of The Institution of Engineers (India): Series C	2021	China	China	1	Comparison of PLA parts meeting different performance requirements	PLA	Cradle to gate, including material transportation	1-sample 27 cm^3 cube with different printing parameters (layer height, infill degree, printing speed and printing temperature)	China Life cycle Basic Database (CLCD)<br>ELCD database<br>Ecoinvent v. 3.0	eFootprint software	Global warming potential (GWP)<br>Primary energy demand (PED)<br>Acidification potential (AP)	According to the orthogonal analysis, infill degree is the most critical parameter that affects the energy consumption in FFF, followed by layer height, printing temperature, and printing speed.<br>When the printing parameters are set to the most energy-saving and most energy-consuming combinations, the energy consumption can differ by nearly 15 times.<br>Increasing the printing speed decreases the energy usage less than expected, because the start-and-stop operations become more frequent, thus taking more energy from the stepper motor, and because the increased material flow requires faster heating.<br>Energy consumption in an FFF printer is due to electric heating (nozzle and plate), stepper motor (3 axes + extruder), microcontroller operation and cooling fan.<br>Electric heating is the largest energy-consuming part, followed by stepper motors.<br>The performance of FFF parts can be modulated through the printing parameters. This may avoid overperformance, however, this may also reduce the service life of FFF parts. Also, the performance must remain comparable to conventional parts.<br>While changing the layer height only affects the 3D printing process, the infill degree will affect both the energy required for printing, and the feedstock production.	Transportation of pellets and transportation of filaments are combined in one process.<br>Impact associated with the production of mechanical equipment not considered (as per Chen et al., Biores. Technol. 2012;114:357–64).<br>Material waste is not considered.
Mecheter A., Tarlochan F.	Fused filament fabrication three-dimensional printing: Assessing the influence of geometric complexity and process parameters on snergy and the environment	Sustainability	2023	Qatar	Qatar	1	Assessment of role of part's complexity, and material and printing parameters for given geometry in education	PLA<br>Tough (T-) PLA<br>ABS	Cradle to gate	Gears: 5 models of different complexity;<br>Unit for LCA and CED: spur gear corresponding to 3 g	Ecoinvent v. 3.8		ReCiPe, Egalitarian, Midpoints;<br>Cumulative energy demand	The geometric complexity had a minimal impact on the energy consumption of the ABS and T-PLA materials. However, for the PLA parts, there was a slight increase in energy consumption with higher geometric complexity;<br>Printing parameters:<br>A noticeable increase in energy consumption can be observed as the layer height decreases for all materials;<br>As the infill density increases, more material is used, which leads to longer printing times and, consequently, higher energy consumption;<br>the choice of infill pattern does not have a significant effect on energy consumption;<br>the change in energy consumption due to temperature variation is insignificant;<br>nonetheless, PLA exhibits the lowest energy values, due to its low melting point;<br>higher printing speeds correspond to lower energy consumption<br>PLA has a lower carbon footprint throughout its entire life cycle compared to ABS.	Unknown toughening mechanism for T-PLA.<br>Parameters are based on the UltiMaker printer model adopted in this study, but they may require fine-tuning depending on the specific material, filament, machine model, and the desired print quality.
Role of different feedstock materials															
Bay C., Nagengast N., Schmidt H.-W., Döpper F., Neuber C.	Environmental assessment of recycled petroleum and bio based additively manufactured parts via LCA	Lecture Notes in Mechanical Engineering	2023	Germany	Germany<br>Germany	1	Comparison of FFF parts made of different plastics	bio-based internationally produced PLA + support<br>petroleum based locally produced PP + support	Manufacturing to recycling	Cuboid<br>Impeller	Ecoinvent v. 3.1	SimaPro	ReCiPe, Endpoints<br>Impact 2002 + LCIA method for credibility reference	Higher impact of PLA, mainly due to the drying step before extruding the filament.	Lab-based recycling focuses on performance, rather than resource efficiency. Conversely, industrial scale recycling follows an economical focus with large-sized machinery.
Bianchi I., Forcellese A., Gentili S., Greco L., Simoncini M.	Comparison between the mechanical properties and environmental impacts of 3D printed synthetic and bio-based composites	Procedia CIRP (29th CIRP Life Cycle Engineering Conference)	2022	Italy	Italy	1	LCA and tensile testing for comparison of different composites in FFF	Short hemp-fibre reinforced PLA (HempPLA) by 20 wt.%<br>Short glass-fibre reinforced PA (GlassPA) by 30 wt.%	Cradle to gate	The production of a tensile specimen with a length of 170 mm, that shows a strain equal to 0.6% when subjected to a tensile load of 1.36 kN	Ecoinvent v. 3.1	SimaPro 9.1.0.11	Global Warming Potential (GWP), according to IPCC (Intergovernmental Panel in Climate Change) 100a methodology;<br>Cumulative Energy Demand (CED).<br>Sensitivity analysis based on the functional unit defined as a tensile specimen according to ASTM D3039	The synthetic composite parts showed significantly better tensile performance (esp. UTS) than the bio-based counterparts.<br>Despite the greater quantity of fibres and matrix being used in the functional unit, HempPLA produces lower GWP and CED than GlassPA.<br>The main difference between the two scenarios is determined by the heating and drying phases that are not present for the HempPLA filament production (not by HempPLA being bio-based as opposed to the synthetic GlassPA).<br>When components with the same shape and size are compared, the environmental benefits related to HempPLA become more noticeable.<br>Having lower density, HempPLA is ideal for lightweight non-structural applications.	Potential improvements:<br>- Different combinations of matrix and fibres should be considered;<br>- Different weight fractions should also be examined;<br>- The LCA should be extended to account for usage and disposal stages;<br>- Specific components, for example in the automotive field, may be conveniently modelled.

Bianchi I., Forcellese A., Mancia T., Simoncini M., Vita A.	Process parameters effect on environmental sustainability of composites FFF technology	Materials and Manufacturing Processes	2022	Italy	Italy	1	Comparison of different composites under different loading conditions	Short carbon-fibre reinforced PA (CarbonPA)<br>Short glass-fibre reinforced PA (GlassPA)	Cradle to grave, but not usage (transportation?) (i.e., cradle to gate plus disposal)	The production of a tensile specimen that exhibits a maximum strain equal to 2.55% when subjected to a tensile load of 3.1 kN and has a length of 170 mm, corresponding to a part of 18.21 g for GlassPA, and 9.21 g for CarbonPA	Ecoinvent v. 3.1	SimaPro 9.1.0.11	Cumulative Energy Demand;<br>Global Warming Potential.<br>Sensitivity analysis based on the functional unit for flexural tests defined "as the production of a 3D printed sample that, during a flexural test, exhibits a maximum strain of 2% when subjected to a load of 556.8 N and has a length of 100 mm"	Pre-assessment of printing parameters as speed, extrusion temperature, and layer thickness, that compromise low energy consumption, good mechanical properties, and nice aesthetics.<br>CarbonPA is characterised by the lowest environmental impact for both endpoints (the energy consumptions of heating and printing phases have a linear relationship with the required printing time and, therefore, with the part's weight).<br>due to its high weight fraction, PA production represents the main contribution on the total impacts of the filaments.<br>CED and a GWP of glass fibres are much lower than carbon fibres.<br>If samples with the same flexural behaviour are considered, the weight saving obtained by using CarbonPA is not very significant (about 11%), and now CarbonPA has the highest environmental impacts due to the raw materials (carbon fibres).<br>Finally, the environmental friendliness of one material over another depends on the specific application and the operating loads that the components must withstand.	Different volume fraction of fibres in different commercial filaments
Fico D., Rizzo D., De Carolis V., Montagna F., Palumbo E., Corcione C.E.	Development and characterization of sustainable PLA/Olive wood waste composites for rehabilitation applications using Fused Filament Fabrication (FFF)	Journal of Building Engineering	2022	Italy	Italy	1	Comparison of different PLA composites with olive wood waste	PLA + olive wood waste	Cradle to gate (A1-A3) with options (A4-transport stage)	To print a 3D ornamental element by FFF with a volume of 8000 cm^3<br>.	Ecoinvent v. 3.8	SimaPro 9.3.0.3	Impact indicators as provided by CEN TC 350;<br>Indicator results normalized with the CML (baseline) normalisation factors (CML-IA Characterization Factors v. 4.2) for the territorial unit EU25 +3	PLA is a renewable material and wood waste is easy to find.<br>The production of PLA granules, followed by grinding and drying, are the largest contributors towards GWP and AP-Fossil fuel for all materials.<br>Due to the presence of wood, energy consumption for extruding and printing increases over neat PLA.<br>However, the total impact decreases by 5.5%, for 10 wt.% of wood, and by 10%, for 20 wt.% of wood.	Due to the high porosity of the composite parts, the wood-filled filaments are unsuitable for building/structural applications
Recycling															
Chatzipanagiotou K.-R., Antypa D., Petrakli F., Karatza A., Pikoń K., Bogacka M., Poranek N., Werle S., Amanatides E., Mataras D., Koumoulos E.P.	Life cycle assessment of composites additive manufacturing using recycled materials	Sustainability	2023	Belgium	Belgium	3	Comparison of FFF composites with virgin fibres vs. recycled fibres via solvolysis	Short carbon-fibre (15 wt.%) reinforced nylon	Cradle to grave, but not usage (transportation?) (i.e., cradle to gate plus disposal)	Coupon (ASTM D638 Type I) with a total weight of 9 g, "ideal" lifetime of 5 years, required service time of 10 years	Ecoinvent v. 3.8	SimaPro 9.4.0.2	Midpoints<br>Environmental Footprint (EF) 3.0 (version 1.03)	Key-point: Fibre recovery is likely to cause fibres to degrade. The functionality loss, which depends on the recovery process, governs the product's lifetime.<br>Impact of two different solvolysis processes for the recovery of carbon fibres (CFs):<br>- As end-of-life treatment, compared to landfilling: both solvolysis treatments result in negative environmental impacts across most impact categories, given that the recovered product (i.e., CFs) can be credited as an avoided product;<br>- As secondary process yielding fibres: even with an assumed 50% loss of functionality, a significant decrease in EIs in most impact categories was calculated for the two solvolysis processes, compared to the manufacture of virgin CFs;<br>- Between solvolysis processes: supercritical solvolysis has the lowest impact across all impact categories; plasma-enhanced solvolysis has a lower impact compared to conventional processes in all impact categories except for ionizing radiation.<br>- in the FFF of composites: products undergoing solvolysis and receiving recovered fibres had a lower impact than the baseline product, even when the fibres lost 50% of their functionality, apart from ionizing radiation (and, to a minor extent, freshwater eutrophication).<br>The EI of FFF can be improved by printing multiple products in the same job, thus decreasing the electricity consumption allocated to each product during the warm-up phase of the equipment and resulting in lower filament loss per product.	As stated by the Authors:<br>- The reason for ionizing radiation is Radon-222, originating from nuclear power production included in the Ecoinvent market group for medium-voltage electricity within the geography "Europe without Switzerland".<br>- The processes of sizing and manufacturing the reinforced filament were excluded, as a sufficiently detailed inventory could not be retrieved from the literature to model them.<br>- Analysis applies to lab-scale solvolysis processes, which need optimisation and scale-up.<br>- The LCA should be accompanied by technical and economic assessments.<br>- More meaningful functional units should be considered once more accurate data becomes available.
Ragab A., Elazhary R., Schmauder S., Ramzy A.	Recycled polyethylene terephthalate/high-density polyethylene sustainability	Sustainability	2023	Egypt	Egypt	2	Comparison of functionalised blends of recycled polymers vs. virgin material blend and unfunctionalised recycled blend	80:20 blends of recycled OET and recycled PP, functionalised (2 methods) and reinforced with 10 wt.% chopped glass fibers	1 kg of each material	Cradle to gate for producing the filament, not for printing (excluding transport and storage logistics)	Ecoinvent v. 3<br>Literature for PET recycling<br>Experimental data wherever possible	Umberto LCA	ReCiPe, Midpoints	Both blends (with compatibilisation with maleic anhydride, GMA, or with surface functionalisation of PET with sodium dodecyl sulphate, SDS) would be more economical to use than commercially available spools of similar materials.<br>Since it was assumed that extrusion would be the same for every filament regardless of its composition, energy consumption for manufacturing, which is the main reason for EI, is the same for all. However, the (recycled) R–Control blend is composed of fewer (recycled) materials and has lower impact in the raw material phase. Amongst recycled blends, SDS–GF has the highest contribution from the raw materials phase due to the presence of reinforcing glass fibres (and compatibilisation strategy?)<br>The (virgin) V–control blend shows the highest environmental impact cumulatively.	
Yip W.C., Yusuf Y., Mastura M.T.	Life cycle analysis (LCA) using CES-Edupack software of new wood dust reinforced recycled prolypropylene composite filament for fused deposition modelling (FDM)	Lecture Notes in Mechanical Engineering	2022	Malaysia	Malaysia	1	Comparison of composite filament produced with recycled PP and wood dust	PP<br>ABS<br>Wood dust				CES-Edupack software with Eco Audit Tool application	Identification of life stages that consume the most energy and produce the most CO_2;<br>water consumption;<br>toxicity factor;<br>sustainability factor	Recycled PP and wood dust (waste) have the potential to be utilised as FFF materials.<br>Recycling PP is a recovering process of the materials that are melted in high temperature and turned into something useful through particular processes such as FFF.	
Zhao P., Rao C., Gu F., Sharmin N., Fu J.	Close-looped recycling of polylactic acid used in 3D printing: An experimental investigation and life cycle assessment	Journal of Cleaner Production	2018	China	China	1	Comparison of PLA close-looped recycling vs. landfilling and incineration	PLA	End-of-life disposal	1 kg of 3D printable PLA	GaBi software database	GaBi ts	ReCiPe, Midpoints, top-down approach, normalised	Close-looped recycling (through substitution of virgin PLA with recycled PLA for producing printable filament) and incineration (through electric power coming from recuperated (heat) energy) can achieve significant environmental savings in the selected impact categories, whereas landfill only results in environmental burdens.<br>Recycling is most favourable in the freshwater eutrophication potential (FEP) category, as it avoids substantial water consumption for PLA polymerisation.<br>Incineration has benefits for terrestrial acidification potential (TAP) and ozone depletion potential (ODP) because generation of electric power in China still largely relies on coal firing.<br>Among other impacts, landfill is associated with substantial release of persistent organic pollutants (POPs) and particles.<br>Additional benefits of recycling:<br>- meets stringent environmental legislation, for example Extended Producer Responsibility in China that requires the use of recycled materials in manufacturing new products and the stipulated proportion of recycled materials is set at 20 wt.%;<br>- from the economic perspective, only requires energy cost of shredding, drying and extruding (estimated as less than 1 USD for reprocessing 1 kg PLA), which is far less than the cost of virgin PLA pellets (18 USD per 1 kg virgin PLA;<br>- enables fast processing speed.	Due to the decay in rheology that prevents further printing, PLA was assumed to be close-looped recyclable only once<br>Blends of virgin and recycled PLA were not accounted for<br>Transportation was neglected, as comparing disposal procedures was the only scope of LCA
